# TGFB2 as a Prognostic Biomarker Associated with Myeloid-Enriched, Multi-Checkpoint-Activated Immunosuppression in Diffuse Glioma: A Multi-Cohort Transcriptomic Study

**DOI:** 10.3390/cancers18132092

**Published:** 2026-06-27

**Authors:** Ehab Balawi, Zhicheng Jiang, Xianwei Wang, Dong Chen

**Affiliations:** 1 Graduate School, Dalian Medical University, Dalian 116044, China; 2 Department of Neurosurgery, Dalian Municipal Central Hospital, Dalian 116033, China; jiangzhicheng@mail.dlut.edu.cn (Z.J.);; 3 Faculty of Medicine, Dalian University of Technology, Dalian 116024, China

**Keywords:** TGFB2, diffuse glioma, microglia, immune checkpoint, immune evasion, tumor microenvironment

## Abstract

**Background/Objectives**: TGFB2 is the dominant TGF-β isoform in glioma, and isolated experimental studies have implicated it in immunosuppressive signaling; however, its prognostic value and systematic association with the tumor immune microenvironment across the diffuse glioma spectrum have not been comprehensively characterized in large clinical cohorts. **Methods**: A multi-cohort transcriptomic study was conducted using TCGA (*n* = 667) as discovery and CGGA (*n* = 404) as validation, integrating survival analysis, functional enrichment, immune deconvolution by ssGSEA and MCP-counter, immune checkpoint correlation, and TISCH2-based single-cell localization. **Results**: TGFB2 was consistently overexpressed in glioma relative to normal brain at both mRNA and protein levels, with expression highest in GBM (median 10.60 vs. 8.45 in LGG; *p* < 2.2 × 10^−16^) and increasing across WHO grade. High TGFB2 predicted worse overall survival in both cohorts (TCGA: 648 vs. 2907 days; CGGA: 863 vs. 3107 days; both *p* < 0.0001), with 3-year AUCs of 0.823 and 0.714, and retained independent prognostic significance in the CGGA multivariate model (HR = 1.343; *p* = 3.2 × 10^−4^). Hallmark GSEA identified consistent enrichment of interferon signaling, epithelial–mesenchymal transition, TNFα/NF-κB, and IL-6/JAK/STAT3 pathways. ssGSEA and MCP-counter concordantly demonstrated significantly expanded myeloid, monocytic, and stromal populations across both cohorts. TGFB2 correlated positively with PD-L1, TIM-3, ICOS (ρ = 0.449), IL2RA (ρ = 0.397), CTLA4 (ρ = 0.375), and TIGIT (ρ = 0.170) in TCGA, with all associations replicated in CGGA. **Conclusions**: TGFB2 is an adverse prognostic biomarker in diffuse glioma coupled to a myeloid-enriched, multi-checkpoint-activated tumor microenvironment, supporting its evaluation as a stratification biomarker in TGF-β/checkpoint combination immunotherapy trials.

## Simple Summary

Glioma is the most common and deadly primary brain tumor in adults, and current treatments, including surgery, radiotherapy, and chemotherapy, rarely cure the disease. The immune system should be able to attack tumor cells, but in glioma it is actively suppressed, preventing immune therapies from working. In this study, we investigated a protein called TGFB2, which is known to suppress immune responses. We found that brain tumors with high TGFB2 levels consistently showed an accumulation of immune-suppressing cells, simultaneous activation of multiple immune checkpoint pathways that block anti-tumor immunity, and worse patient survival across two large independent patient cohorts. These findings suggest that TGFB2 is closely linked to the immune-suppressive environment of brain tumors and may help identify patients most likely to benefit from therapies that combine TGFB2 blockade with immune checkpoint inhibitors.

## 1. Introduction

Diffuse gliomas are the most common and lethal primary central nervous system tumors in adults [1,2], encompassing a biologically heterogeneous spectrum that ranges from slow-growing isocitrate dehydrogenase (IDH)-mutant oligodendrogliomas and astrocytomas to rapidly fatal IDH-wildtype glioblastoma [2]. Globally, brain and CNS cancers accounted for 321,624 new cases and 248,403 deaths in 2022 (age-standardized incidence rate 3.5 per 100,000) [3], with gliomas representing the majority of malignant CNS tumors [4]. In China, a multicenter cross-sectional study estimated the age-standardized prevalence of primary brain tumors at 22.52 per 100,000, with gliomas accounting for 55.56% of cases in individuals aged 0–19 years and 31.10% in those aged 20–59 years [5]. More recent national burden estimates reported 105,541 incident and 305,063 prevalent malignant brain and CNS cancer cases in China in 2021 (age-standardized incidence rate 6.12 per 100,000) [6], and contemporary registry data (2019–2020) confirm that gliomas remain the dominant malignant subtype, with glioblastoma, astrocytoma, and oligodendroglioma collectively accounting for over 70% of malignant cases [7]. The etiology of diffuse glioma remains incompletely understood. Established risk factors are largely confined to ionizing radiation exposure and rare inherited cancer predisposition syndromes, with no other environmental or behavioral exposure consistently established as causal [8]. Genome-wide association studies have further identified susceptibility loci implicating genes such as *TERT*, *EGFR*, and *CDKN2A*/*CDKN2B*, although the genetic architecture of glioma risk differs appreciably between East Asian and White populations [9,10].

Despite multimodal therapy comprising maximal safe surgical resection, radiotherapy, and temozolomide chemotherapy [11], median overall survival in glioblastoma remains approximately 15 months, and five-year survival rates fall below 10% [1,12]. Immunotherapy has transformed outcomes in several solid tumors, motivating substantial interest in its application to glioma; however, this potential has not yet translated into meaningful clinical benefit in glioblastoma, underscoring the need to better characterize the molecular determinants of the glioma immune microenvironment [13]. The fifth edition of the World Health Organization Classification of Tumors of the Central Nervous System (WHO CNS5, 2021) transformed glioma taxonomy by integrating molecular markers, principally IDH mutation status, 1p/19q codeletion, and CDKN2A/B deletion, as primary diagnostic and prognostic determinants alongside histological grade [14]. This molecular framework has sharpened biological stratification but has not yet been matched by therapeutic advances, emphasizing the importance of discovering new prognostic biomarkers and immune-related therapeutic targets that operate within and across these molecularly defined entities.

Transforming growth factor-β (TGF-β) is a pleiotropic cytokine belonging to a large superfamily of structurally related growth factors [15,16]. It signals through a canonical SMAD-dependent pathway and through multiple non-canonical SMAD-independent cascades, including PI3K/AKT, MAPK/ERK, RHO-GTPase, NF-κB, and JAK/STAT signaling. In the canonical pathway, ligand binding to TGF-β receptor II (TβRII) recruits and transphosphorylates TβRI (ALK5), activating SMAD2/3 to form a complex with SMAD4 that translocates to the nucleus and functions as a transcriptional regulator [15,16]. The three mammalian TGF-β isoforms (TGF-β1, TGF-β2, and TGF-β3) are structurally related but exhibit distinct receptor-binding affinities and differential tissue distribution patterns [16]. TGF-β2 binds poorly to TβRII alone and requires TGF-β receptor III (TβRIII, β-glycan) as a co-receptor to achieve high-affinity receptor engagement [16], conferring distinct biological properties from the other isoforms. Under physiological conditions, TGF-β acts as a growth suppressor by promoting cell cycle arrest and triggering apoptotic programs; however, in established malignancies, tumors acquire resistance to its growth-inhibitory effects while retaining and amplifying its pro-tumorigenic outputs, a well-characterized phenomenon termed the TGF-β paradox [15,17].

In glioma, TGF-β2 is the principal isoform and was originally identified as “glioblastoma-derived T-cell suppressor factor,” reflecting its potent capacity to impair cytotoxic T-lymphocyte function and promote immune evasion [18]. Elevated TGF-β2 expression correlates with increasing histological grade and has been experimentally linked to multiple oncogenic programs: induction of glioma stem cell self-renewal through LIF, Sox4–Sox2, and Id1–Id3 signaling [18]; promotion of invasion through matrix metalloproteinase induction and epithelial–mesenchymal transition (EMT) [18,19]; angiogenesis via vascular endothelial growth factor (VEGF) and IGFBP7 upregulation [18]; and broad immunosuppression through inhibition of natural killer cells, dendritic cells, and cytotoxic T lymphocytes, while simultaneously expanding FoxP3^+^ regulatory T cells and promoting M2 macrophage polarization [18,19]. Given that PD-L1, TIM-3, IDO1, CTLA4, and TIGIT are each individually validated immunotherapeutic targets, identifying upstream regulators that coordinate their simultaneous expression could inform rational combination immunotherapy design in glioma [20]. TGF-β2 also promotes mesenchymal glioblastoma identity, the most aggressive transcriptional subtype, through SMAD-driven activation of EMT transcription factors including SLUG and ZEB1, and recent evidence implicates RUNX2-mediated TGF-β/SMAD signaling as a driver of malignant progression specifically in IDH-wildtype tumors [21].

Despite this compelling biological rationale, clinical translation of TGF-β inhibition in glioma has been uniformly disappointing. Galunisertib (LY2157299), the most extensively evaluated TβRI kinase inhibitor, failed to improve overall survival in both newly diagnosed and recurrent glioblastoma [22,23]. The TGF-β2-targeted antisense oligonucleotide trabedersen was terminated at Phase III for futility despite promising earlier-phase signals in anaplastic astrocytoma [24]. Inadequate blood–brain barrier penetration, absence of biomarker-driven patient selection, and rapid activation of compensatory immunosuppressive pathways have been identified as key mechanisms of failure [25]. These setbacks underscore the necessity for systematic transcriptomic profiling of TGFB2-high glioma to delineate the molecular and immunological landscape in which TGF-β2 operates and to define the patient subpopulations most likely to benefit from targeted therapeutic strategies.

Prognostic biomarkers play an increasingly central role in precision oncology by enabling refined patient risk stratification, earlier and more accurate outcome prediction, and improved therapeutic targeting [26]. Previous studies of TGFB2 in glioma have been predominantly experimental or have focused on single cohorts, individual molecular associations, or isolated clinical endpoints. A comprehensive multi-cohort characterization integrating expression across the molecular glioma taxonomy of WHO CNS5, prognostic analysis with full clinicomolecular adjustment, functional enrichment, immune microenvironment deconvolution, checkpoint co-expression, and single-cell localization has, to our knowledge, not been performed. Here, we addressed this gap using paired discovery (TCGA, *n* = 667) and validation (CGGA, *n* = 404) diffuse glioma cohorts. Analyses encompassed protein-level screening, Hallmark gene set enrichment, single-sample gene set enrichment analysis (ssGSEA) and MCP-counter immune deconvolution, and TISCH2-based single-cell localization, collectively producing a multi-layered transcriptomic portrait of TGFB2 and appraising its candidacy as a prognostically and immunologically informative marker in diffuse glioma.

## 2. Materials and Methods

### 2.1. Study Design

**Figure 1:**
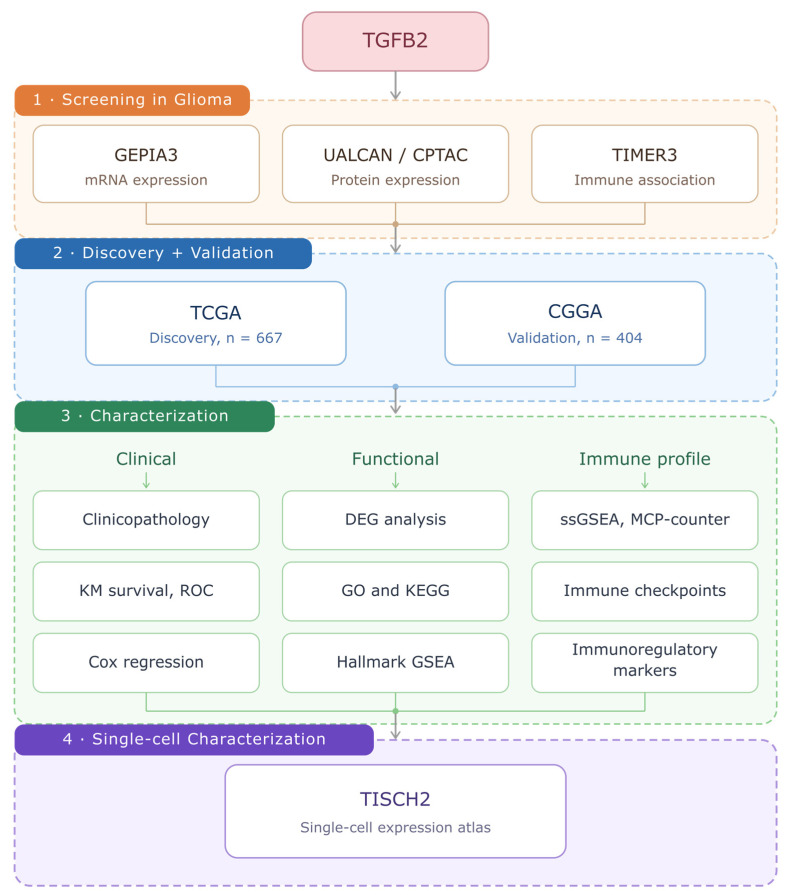
Overview of the study workflow. TGFB2 was evaluated through an initial multi-tool screening layer, followed by comprehensive characterization across clinical, functional, and immune profiling axes in two independent RNA-seq cohorts, with single-cell resolution provided by the TISCH2 atlas.

The present study adopted a staged bioinformatics framework to characterize the expression, prognostic significance, and tumor microenvironmental associations of TGFB2 in diffuse glioma. Exploratory screening was first conducted using public web-based resources to assess differential expression, survival relevance, and immune associations of TGFB2 in glioma. Main cohort analyses were then performed using bulk RNA sequencing (RNA-seq) data from the TCGA [27] discovery cohort, with independent external validation in the CGGA [28] cohort. Finally, publicly available single-cell RNA-seq datasets accessed through TISCH2 [29] were used to contextualize TGFB2 expression at the cellular level within the glioma microenvironment (Figure 1).

### 2.2. Exploratory Screening

Exploratory screening was performed using three public web-based resources to assess differential expression, proteomic expression, prognostic relevance, and immune associations of TGFB2 in glioma prior to main cohort analysis. Tumor-versus-normal mRNA expression and overall survival were evaluated in glioblastoma (GBM) and lower-grade glioma (LGG) using GEPIA3 (https://gepia3.bioinfoliu.com/, accessed 24 February 2026) [30], which integrates TCGA tumor expression data with normal tissue references from GTEx [31]. Protein-level expression in glioblastoma was assessed using the UALCAN (University of ALabama at Birmingham CANcer data analysis) portal (https://ualcan.path.uab.edu, accessed 24 February 2026) [32], which provides CPTAC-based proteomic data for tumor-versus-normal comparisons across cancer types and, including glioblastoma. Immune-context screening was performed using TIMER3 (https://compbio.cn/timer3/, accessed 24 February 2026) [33], with CD8+ T-cell and regulatory T-cell associations evaluated separately in GBM and LGG. All portal-based outputs were treated as hypothesis-generating evidence and were not used as primary analytical findings.

### 2.3. Data Sources

TCGA discovery cohort data, comprising bulk RNA-seq expression matrices, clinical variables, and curated overall survival information, were retrieved from the Genomic Data Commons (GDC) portal (https://gdc.cancer.gov/, accessed 24 February 2026) [34]. Molecular annotation including IDH mutation status, 1p/19q codeletion, and O^6^-methylguanine-DNA methyltransferase (MGMT) promoter methylation status was obtained separately from the cBioPortal PanCancer Atlas (https://www.cbioportal.org/, accessed 24 February 2026) [35,36]: Brain Lower Grade Glioma (TCGA, PanCancer Atlas) and Glioblastoma Multiforme (TCGA, PanCancer Atlas), both corresponding to the TCGA Cell 2018 publication [37,38]. All files were merged by matched sample and patient identifiers, and the cohort was restricted to primary tumors with non-missing overall survival (OS) status and time, yielding a final TCGA discovery cohort of 667 primary glioma cases.

The independent CGGA validation cohort (mRNAseq_693) [28,39,40,41] was retrieved from the CGGA data repository (https://www.cgga.org.cn/download.jsp, accessed 24 February 2026) using the STAR + RSEM Fragments Per Kilobase Million (FPKM) expression matrix and matched clinical data file. The cohort was restricted to primary tumors with non-missing OS status and event data, yielding 404 cases. Throughout all analyses, CGGA FPKM values were subjected to log2(FPKM + 1) transformation prior to use. Full cohort characteristics for both datasets are provided in Table 1.

### 2.4. Expression and Clinicopathological Analyses

TGFB2 expression was compared between LGG and GBM, across WHO grade, and across histological subtype in both the TCGA discovery and CGGA validation cohorts. Between-group statistical comparisons employed the Wilcoxon rank-sum test for two-group contrasts and the Kruskal–Wallis test for multi-group analyses. Associations between TGFB2 expression and molecular variables, specifically IDH mutation status, 1p/19q codeletion, and MGMT promoter methylation status, were additionally evaluated in both cohorts. Continuous TGFB2 expression across molecular subgroups was evaluated by the Wilcoxon rank-sum test; differences in categorical variable distributions were assessed by chi-square testing. For descriptive cohort visualization, samples in each cohort were ordered by increasing TGFB2 expression and displayed with clinicopathological annotation tracks using ComplexHeatmap (v2.26.1) [42]. All boxplot-based visualizations were generated using ggplot2 (v4.0.3) [43].

### 2.5. Survival Analysis

Overall survival (OS) served as the primary endpoint. Cases were eligible for survival analysis only if TGFB2 expression data, OS status, OS time, and a follow-up duration exceeding zero days were all available. In the TCGA cohort, three cases with a recorded follow-up time of zero were excluded from survival analyses, yielding a final survival analysis cohort of 664 cases; the full 667-case cohort was retained for all other analyses.

Each cohort was stratified into TGFB2-high and TGFB2-low subgroups at the cohort-specific median expression value (TCGA: 8.9743; CGGA: 2.245, log2[TGFB2 + 1] scale). The median was chosen as the stratification threshold in preference to optimized data-driven cutoffs, prioritizing cross-cohort reproducibility and reducing the risk of in-sample overfitting. Kaplan–Meier curves were generated to depict survival distributions, with between-group statistical comparison by the log-rank test. Prognostic discrimination of TGFB2 was further quantified by time-dependent receiver operating characteristic (ROC) analysis at 1, 2, and 3 years, with area under the curve (AUC) values derived for each time point.

The independent prognostic contribution of TGFB2 was assessed by Cox proportional hazards regression, with TGFB2 modeled as a continuous variable alongside the following covariates: age at diagnosis, WHO grade, IDH mutation status, MGMT promoter methylation, and 1p/19q codeletion. Variables were first examined in separate univariate models and subsequently entered jointly into a single multivariate model. Results are expressed as hazard ratios (HRs, reflecting the relative risk of death associated with each unit increase in TGFB2 expression) with 95% confidence intervals (CIs) and two-sided *p* values. Multivariate analyses were based on complete-case data owing to missing values in a subset of clinicomolecular variables.

All survival analyses were implemented in R (v4.5.2), with Kaplan–Meier and Cox regression performed via the survival [44] (v3.8-6) and survminer [45] (v0.5.2) packages; time-dependent ROC curves were computed using timeROC [46] (v0.4.1); and visualizations were produced with ggplot2 [43] (v4.0.3).

### 2.6. Differential Expression and Functional Enrichment

Functional enrichment analyses were conducted independently in each cohort, with samples stratified into TGFB2-high and TGFB2-low subgroups as detailed in Section 2.4. Differentially expressed genes were identified using limma [47] (v3.66.0), applying thresholds of Benjamini–Hochberg-adjusted *p* < 0.05 and |log2 fold change| > 1. Gene Ontology (GO) and Kyoto Encyclopedia of Genes and Genomes (KEGG) enrichment analyses were performed on genes upregulated in the TGFB2-high group using clusterProfiler (v4.18.4) [48], following SYMBOL-to-ENTREZ conversion with bitr() using the org.Hs.eg.db annotation database (v3.22.0) [49]. GO enrichment was run via enrichGO() with ontology = “ALL”; the five most significantly enriched terms from each ontology category, biological process (BP), cellular component (CC), and molecular function (MF), were retained for visualization. KEGG enrichment was performed using enrichKEGG() with organism = “hsa”, and the top enriched pathways ranked by adjusted *p* value were retained. Benjamini–Hochberg correction was applied for all multiple testing adjustments throughout.

To complement over-representation analyses and reduce dependence on arbitrary differential expression cutoffs, Hallmark GSEA was conducted on preranked gene lists based on the cohort-specific limma t statistic. Hallmark gene sets were sourced from the msigdbr package [50] (v26.1.0), providing access to the MSigDB Hallmark collection [51], and enrichment was executed via clusterProfiler::GSEA() with Benjamini–Hochberg correction, minGSSize = 10, maxGSSize = 500, and eps = 1 × 10^−10^. Pathways with positive enrichment and adjusted *p* < 0.05 were retained as statistically significant and are reported; full enrichment results are provided in the Supplementary Tables. All enrichment visualizations were generated using ggplot2 (v4.0.3) [43] and enrichplot (v1.30.5) [52].

### 2.7. Immune Infiltration and Checkpoint Analyses

To characterize the tumor immune microenvironment associated with TGFB2, two independent analytical approaches were applied in both cohorts. Immune-cell enrichment was assessed by ssGSEA using a panel of 22 immune-cell signatures (LM22), originally compiled by Newman et al. [53] and accessed via the GSVAdata Bioconductor data package (v1.46.0), implemented via the GSVA (v2.4.9) [54] and GSEABase (v1.72.0) [55] packages. Immune and stromal cell abundance was independently estimated using MCP-counter [56]. In both approaches, TGFB2-high and TGFB2-low groups were defined as described above, and between-group comparisons were evaluated by the Wilcoxon rank-sum test, with Benjamini–Hochberg FDR correction applied for multiple comparisons.

Expression levels of eight immune checkpoint genes (*CD274*, *PDCD1LG2*, *CD276*, *PDCD1*, *CTLA4*, *HAVCR2*, *TIGIT*, and *IDO1*) were extracted from both cohort matrices and contrasted across TGFB2 expression groups by Wilcoxon rank-sum testing. Spearman correlation analysis was performed between TGFB2 and all eight checkpoint genes to characterize the continuous relationship between expression levels, and additionally between TGFB2 and a panel of preselected immunoregulatory markers, *ICOS*, *IL2RA*, *CTLA4*, and *TIGIT*, chosen on the basis of biological relevance to T-cell activation and immune regulation. Only markers showing statistically significant and directionally consistent associations across both cohorts are reported as primary findings. For scatter-plot visualization, expression values were displayed on the log2(x + 1) scale to improve distributional symmetry.

### 2.8. Single-Cell Localization

To investigate the cellular sources underlying the myeloid-enriched microenvironment observed in the bulk cohort analyses, single-cell RNA sequencing datasets were queried through the Tumor Immune Single-cell Hub 2 (TISCH2) database [29], which provides uniformly processed and cell-type-annotated single-cell transcriptomic data across multiple tumor types. Single-cell analyses in TISCH2 were restricted to GBM datasets, as this represents the primary glioma tumor type available with standardized cell-type annotation. The cellular localization findings therefore predominantly reflect the GBM microenvironment. Glioma datasets were screened for detectable TGFB2 expression and major-lineage cell-type annotation encompassing malignant, myeloid, and lymphoid populations. Two datasets meeting these criteria, GSE148842 and GSE84465, were selected for in-depth characterization via UMAP dimensionality reduction embeddings and violin plot visualization. These analyses were intended to provide cellular context for the bulk transcriptomic findings and were not inferential.

### 2.9. Statistical Considerations

Throughout all analyses, statistical tests were two-sided with a significance threshold set at *p* < 0.05. Benjamini–Hochberg FDR correction was applied throughout for all multiple testing adjustments, as described in the relevant sections above. The complete analytical pipeline was implemented in R (v4.5.2) using Bioconductor (v3.22).

## 3. Results

### 3.1. Exploratory Screening Identifies TGFB2 as an Overexpressed and Prognostically Adverse Candidate in Glioma

**Figure 2:**
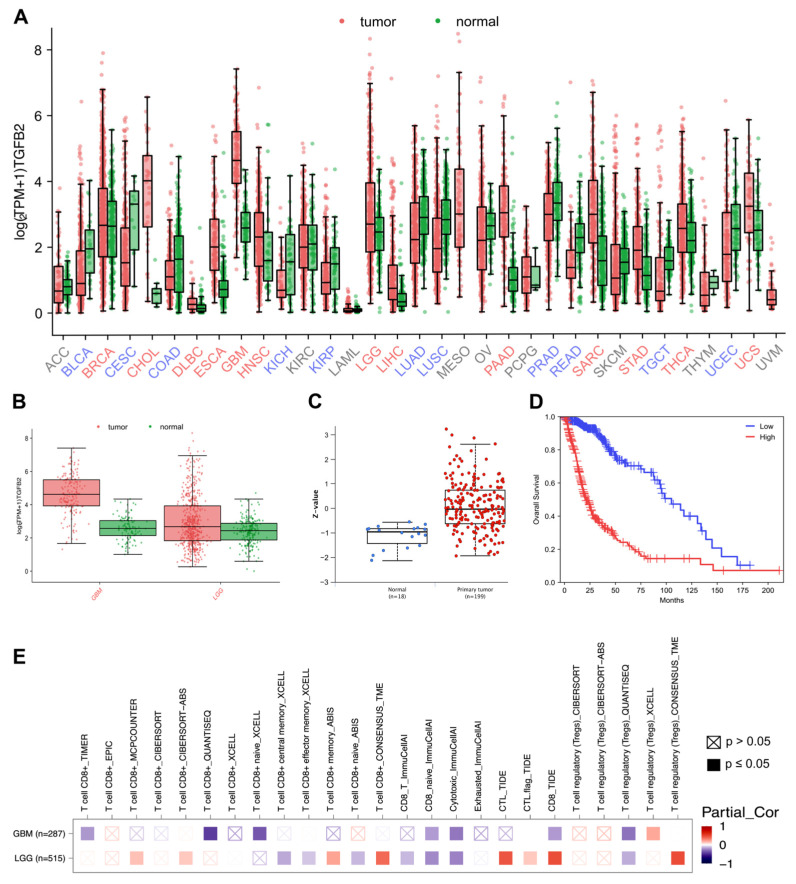
Screening analyses support TGFB2 as an overexpressed, prognostically adverse, and immune-associated candidate in glioma. (**A**) Pan-cancer TGFB2 mRNA expression across tumor and normal tissues from GEPIA3. Cancer type labels in red indicate significantly higher expression in tumor relative to normal, blue labels indicate significantly higher expression in normal relative to tumor, and grey labels indicate no significant difference. (**B**) TGFB2 mRNA expression is significantly elevated in GBM and LGG relative to normal brain tissue. (**C**) TGFB2 protein expression is significantly increased in GBM primary tumors compared with normal brain tissue based on UALCAN/CPTAC proteomic data. (**D**) High TGFB2 expression is associated with significantly worse overall survival in the pooled glioma cohort. (**E**) TIMER3 immune-correlation screening shows variable associations between TGFB2 and CD8+ T-cell and regulatory T-cell signatures across algorithms in GBM and LGG.

Pan-cancer screening via GEPIA3 revealed differential TGFB2 expression across multiple tumor types, with consistent upregulation in both GBM and LGG relative to normal brain tissue (Figure 2A). In glioma-specific analysis, TGFB2 mRNA expression (log2[TPM + 1]) was significantly higher in GBM (median 4.62 vs. 2.57 in normal; *p* = 3.18 × 10^−41^) and in LGG (median 2.68 vs. 2.44; *p* = 1.81 × 10^−7^) compared with normal brain references (Figure 2B). At the protein level, UALCAN/CPTAC proteomic analysis demonstrated that TGFB2 protein expression was significantly higher in GBM primary tumors (median −0.033, *n* = 199) than in normal brain tissue (median −0.951, *n* = 18; *p* = 4.87 × 10^−11^; Figure 2C). Pan-cancer CPTAC proteomic profiling demonstrated variable TGFB2 protein expression across ten tumor types, with glioblastoma showing consistently elevated tumor expression relative to normal tissue (Supplementary Figure S1). In the pooled glioma cohort, exploratory survival screening demonstrated that high TGFB2 expression was associated with markedly inferior overall survival (HR = 4.99, 95% CI 3.70–6.73; *p* = 3.46 × 10^−31^; Figure 2D); this estimate reflects an unadjusted, portal-derived analysis of pooled GBM and LGG without molecular covariate control and substantially overstates the effect size observed in the fully adjusted cohort analyses reported below. TIMER3 immune-context screening revealed variable associations between TGFB2 and CD8+ T-cell and regulatory T-cell signatures across immune estimation algorithms in both GBM and LGG, supporting immune relevance without a uniform directional signal (Figure 2E).

### 3.2. TGFB2 Expression Is Associated with Adverse Molecular and Clinicopathological Features in Glioma

**Figure 3:**
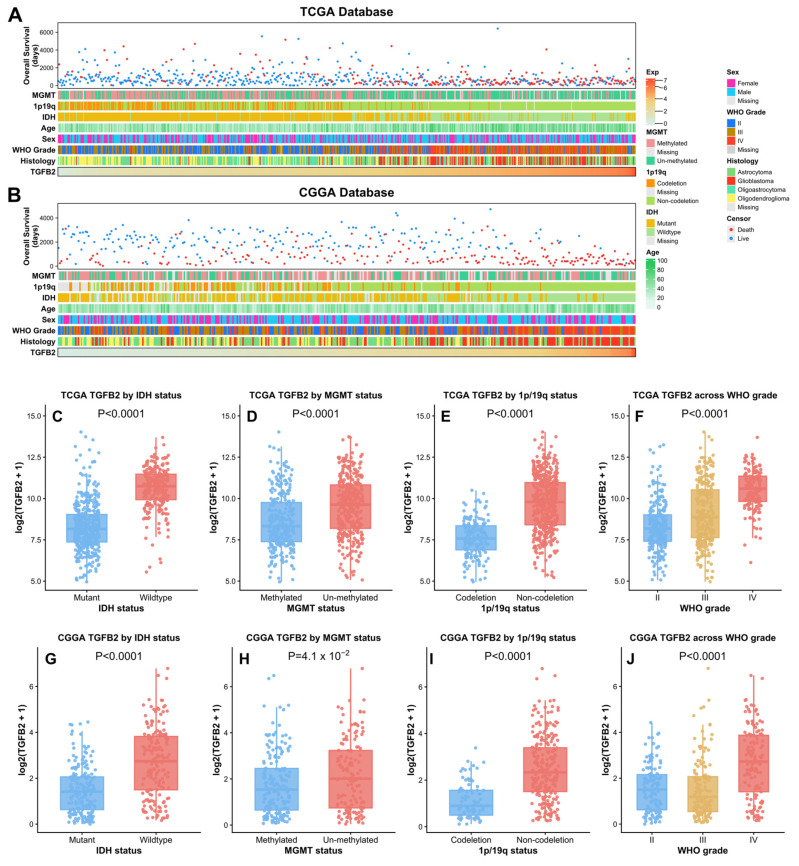
Screening TGFB2 expression is associated with adverse molecular and clinicopathological features in TCGA and CGGA gliomas. (**A**,**B**) Integrated overview panels for the TCGA and CGGA cohorts ordered by increasing TGFB2 expression, displaying sample-wise distributions of overall survival, MGMT promoter methylation, 1p/19q codeletion, IDH status, age, sex, WHO grade, and histology. (**C**–**F**) TGFB2 expression in TCGA according to IDH status, MGMT promoter methylation status, 1p/19q codeletion status, and WHO grade, respectively. (**G**–**J**) Corresponding comparisons in the CGGA cohort. *p* values were calculated using the Wilcoxon rank-sum test for two-group comparisons and the Kruskal–Wallis test for WHO grade comparisons. CGGA expression values are shown as log2(TGFB2 + 1).

Integrated clinicopathological overview panels demonstrated that increasing TGFB2 expression tracked consistently with adverse tumor characteristics in both cohorts, with visually evident enrichment in IDH-wildtype, non-codeleted, and higher-grade tumors (Figure 3A,B).

In the TCGA discovery cohort, the strongest and most biologically meaningful associations were observed for molecular markers. TGFB2 expression was significantly higher in IDH-wildtype than IDH-mutant tumors (*p* < 0.0001; Figure 3C), higher in MGMT promoter-unmethylated than methylated tumors (*p* < 0.0001; Figure 3D), and higher in non-codeleted than codeleted tumors (*p* < 0.0001; Figure 3E). These molecular associations are consistent with the biological hierarchy established by the 2021 WHO Classification of CNS Tumors [2,14], in which IDH mutation status and 1p/19q codeletion define the principal glioma entities. TGFB2 was also significantly higher in GBM than in LGG (median 10.60 vs. 8.45, Wilcoxon *p* < 2.2 × 10^−16^; Supplementary Figure S2A) and differed significantly across WHO grades (Kruskal–Wallis χ^2^ = 154.87, *p* < 2.2 × 10^−16^), with the highest levels in grade IV tumors (Figure 3F). Histology-based analysis showed the highest expression in glioblastoma and the lowest in oligodendroglioma (Supplementary Figure S2B).

The same molecular-first pattern was validated in the CGGA cohort. TGFB2 remained significantly higher in IDH-wildtype tumors (*p* < 0.0001; Figure 3G), whereas the association with MGMT promoter methylation was weaker but statistically significant (*p* = 0.041; Figure 3H), and TGFB2 was higher in non-codeleted than codeleted tumors (*p* < 0.0001; Figure 3I). TGFB2 was significantly higher in GBM than in LGG (median log2[TGFB2 + 1] 2.73 vs. 1.40, Wilcoxon *p* = 8.643 × 10^−13^; Supplementary Figure S2C) and differed significantly across WHO grades (Kruskal–Wallis χ^2^ = 51.302, *p* = 7.244 × 10^−12^), driven predominantly by markedly higher expression in grade IV tumors with no clear linear progression between grades II and III (Figure 3J). Histology-based analysis again showed the highest expression in glioblastoma and the lowest in oligoastrocytoma and oligodendroglioma (Supplementary Figure S2D).

Collectively, these results demonstrate that TGFB2 overexpression reproducibly co-segregates with the molecularly aggressive glioma phenotype across both cohorts, with IDH-wildtype status, 1p/19q non-codeletion, and advanced WHO grade representing the most consistent correlates.

### 3.3. TGFB2 Is an Adverse Prognostic Marker in Diffuse Glioma

**Figure 4:**
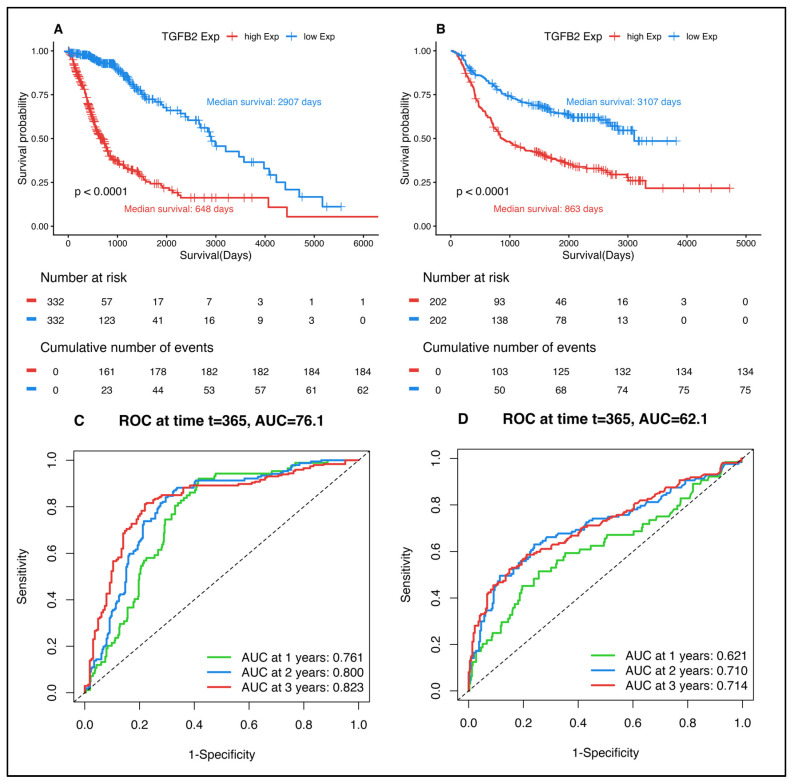
Prognostic value of TGFB2 in diffuse glioma. (**A**,**B**) Kaplan–Meier overall survival curves for TGFB2-high and TGFB2-low groups in the TCGA and CGGA glioma cohorts, respectively. Patients were dichotomized using the cohort-specific median TGFB2 expression. *p* values were calculated using the log-rank test. (**C**,**D**) Time-dependent ROC curves for TGFB2-based prediction of 1-, 2-, and 3-year overall survival in the TCGA and CGGA cohorts, respectively. AUC values are displayed for each time point.

Kaplan–Meier survival curves revealed a consistent and significant adverse survival impact of high TGFB2 expression across both the TCGA discovery and CGGA validation cohorts (Figure 4A,B). In TCGA (*n* = 664), patients in the high-expression group had a median overall survival of 648 days compared with 2907 days in the low-expression group (log-rank *p* < 0.0001). In CGGA (*n* = 404), the corresponding median overall survival values were 863 and 3107 days, respectively (log-rank *p* < 0.0001).

Time-dependent ROC analysis corroborated the prognostic utility of TGFB2 (Figure 4C,D). In TCGA, AUC values at 1, 2, and 3 years were 0.761, 0.800, and 0.823, respectively, indicative of good discriminatory performance. In CGGA, the corresponding values were 0.621, 0.710, and 0.714, confirming moderate but reproducible predictive capacity in the independent validation cohort (Table 2).

**Figure 5:**
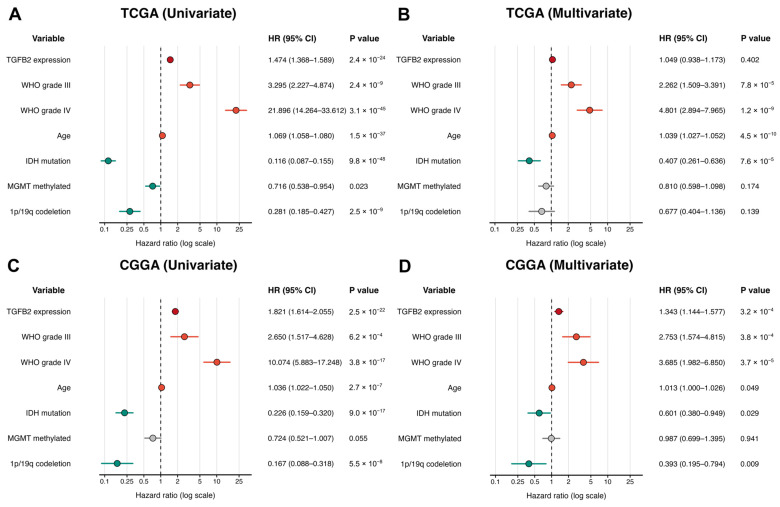
Univariate and multivariate Cox regression analyses of overall survival in the TCGA and CGGA glioma cohorts. (**A**) TCGA univariate analysis. (**B**) TCGA multivariate analysis. (**C**) CGGA univariate analysis. (**D**) CGGA multivariate analysis. Hazard ratios (HRs) and 95% confidence intervals (CIs) are shown. The dashed vertical line indicates HR = 1.

Univariate Cox regression established an adverse prognostic association for TGFB2 in both cohorts, with each unit increase in expression conferring significantly elevated mortality risk in TCGA (HR = 1.474, 95% CI 1.368–1.589; *p* = 2.4 × 10^−24^) and CGGA (HR = 1.821, 95% CI 1.614–2.055; *p* = 2.5 × 10^−22^). In TCGA, this association was accompanied by higher WHO grade, older age, IDH-wildtype status, MGMT promoter unmethylation, and 1p/19q non-codeletion (Figure 5A; Table 3). The corresponding CGGA univariate results are shown in Figure 5C.

In the TCGA multivariate model, after adjustment for age, WHO grade, IDH status, MGMT methylation, and 1p/19q codeletion, TGFB2 did not retain independent prognostic significance (HR = 1.049, 95% CI 0.938–1.173; *p* = 0.402; Figure 5B). In this adjusted model, the dominant independent prognostic factors were WHO grade IV (HR = 4.801, 95% CI 2.894–7.965; *p* = 1.2 × 10^−9^), WHO grade III (HR = 2.262, 95% CI 1.509–3.391; *p* = 7.8 × 10^−5^), age (HR = 1.039; *p* = 4.5 × 10^−10^), and IDH mutation (HR = 0.407; *p* = 7.6 × 10^−5^). In the CGGA multivariate model, however, TGFB2 retained independent adverse prognostic significance following full covariate adjustment (HR = 1.343, 95% CI 1.144–1.577; *p* = 3.2 × 10^−4^; Figure 5D; Table 4), together with WHO grade, age, IDH mutation status, and 1p/19q codeletion.

### 3.4. TGFB2-High Gliomas Harbor Inflammatory, Mesenchymal, and Stress-Adaptive Transcriptomic Programs

The biological programs associated with elevated TGFB2 expression were characterized by differential expression and functional enrichment analyses conducted independently in the TCGA discovery and CGGA validation cohorts.

In TCGA, 2544 genes were significantly differentially expressed between TGFB2-high and TGFB2-low tumors, whereas 11,971 significant genes were identified in CGGA (adjusted *p* < 0.05, |log2 fold change| > 1). The larger number of significant differentially expressed genes (DEGs) identified in CGGA likely reflects the wider dynamic range of FPKM-based expression values relative to the normalized TCGA matrix, rather than a true biological difference in effect size.

GO enrichment of upregulated genes in the TCGA discovery cohort was dominated by immune-regulatory and cell-interaction processes, including leukocyte cell–cell adhesion (Count = 127, adjusted *p* = 9.20 × 10^−23^), regulation of leukocyte cell–cell adhesion (*n* = 114, adjusted *p* = 9.30 × 10^−20^), regulation of T-cell activation (*n* = 114, adjusted *p* = 1.22 × 10^−19^), and regulation of immune effector processes (*n* = 116, adjusted *p* = 3.29 × 10^−19^; Supplementary Figure S3A). In the CGGA validation cohort, GO terms were dominated by nucleocytoplasmic transport (Count = 268, adjusted *p* = 4.28 × 10^−23^), macroautophagy (*n* = 300, adjusted *p* = 1.12 × 10^−22^), RNA splicing (*n* = 351, adjusted *p* = 8.26 × 10^−22^), and Golgi vesicle transport (*n* = 245, adjusted *p* = 1.20 × 10^−21^; Supplementary Figure S3B). The divergence in dominant GO terms between cohorts may partly reflect differences in cohort composition and expression quantification platforms, rather than representing fundamentally distinct biology.

KEGG analysis in TCGA further highlighted cell adhesion molecule interaction, hematopoietic cell lineage, and multiple immune and inflammatory modules (Supplementary Figure S4A). Notably, several enriched KEGG terms, including allograft rejection, graft-versus-host disease, rheumatoid arthritis, and infectious disease pathways, reflect shared immune-response gene modules rather than literal disease associations and should be interpreted accordingly. Similarly, terms such as nicotine addiction and neuroactive ligand signaling reflect overlapping neurotransmitter receptor gene sets rather than neurological biology. KEGG analysis in CGGA further highlighted protein processing in the endoplasmic reticulum, cell cycle regulation, ubiquitin-mediated proteolysis, focal adhesion, autophagy, and endocytosis as the leading enriched pathways (Supplementary Figure S4B).

**Figure 6:**
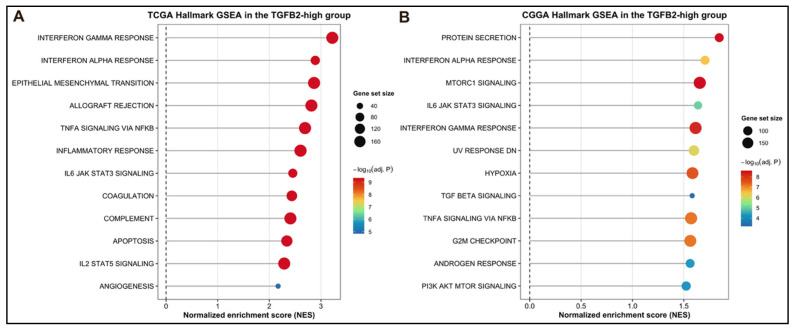
Hallmark gene set enrichment analysis (GSEA) showing significantly positively enriched pathways in TGFB2-high tumors in the TCGA (**A**) and CGGA (**B**) cohorts. Only pathways meeting adjusted *p* < 0.05 in the positive enrichment direction are displayed. Bubble size represents gene count and bubble color represents −log10-adjusted *p* value.

Hallmark GSEA refined this pathway-level biology. The strongest positively enriched programs in TCGA were interferon-γ response (normalized enrichment score, NES = 3.220), interferon-α response (NES = 2.891), epithelial–mesenchymal transition (NES = 2.867), allograft rejection (NES = 2.815), TNF-α signaling via nuclear factor kappa B (NF-κB) (NES = 2.692), inflammatory response (NES = 2.604), and IL-6/JAK/STAT3 signaling (NES = 2.455; Figure 6A). Additional significant positive enrichment was observed for coagulation, complement, angiogenesis, hypoxia, mTORC1 signaling, and glycolysis (Supplementary Table S1). In the negative enrichment direction, only Hedgehog signaling reached statistical significance in TCGA (NES = −1.802, adjusted *p* = 0.006), with no counterpart in CGGA; this isolated finding was not pursued further. In the CGGA validation cohort, Hallmark GSEA identified protein secretion (NES = 1.845), interferon-α response (NES = 1.707), mTORC1 signaling (NES = 1.655), IL-6/JAK/STAT3 signaling (NES = 1.639), interferon-γ response (NES = 1.615), hypoxia (NES = 1.584), TGF-β signaling (NES = 1.582), and TNF-α signaling via NF-κB (NES = 1.571) among the leading positively enriched pathways (Figure 6B). Notably, the enrichment of TGF-β signaling specifically in CGGA TGFB2-high tumors offers direct pathway-level corroboration of the biological centrality of TGFB2 in this setting (Supplementary Table S2).

Although the dominant annotations differed between cohorts, with TCGA emphasizing immune regulation and extracellular interaction and CGGA highlighting secretory, proteostatic, and transport programs, both datasets consistently supported a TGFB2-high state enriched for inflammatory signaling, mesenchymal remodeling, stress adaptation, and tumor–microenvironment interaction. The predominance of positive over negative enrichment across both cohorts, with no significant negatively enriched Hallmark programs in CGGA and only Hedgehog signaling negatively enriched in TCGA, is consistent with TGFB2-high gliomas representing a broadly activated rather than a transcriptionally suppressed tumor state [57,58], strengthening the interpretability of the identified biological programs.

### 3.5. TGFB2-High Tumors Show Concordant Myeloid and Stromal Microenvironmental Enrichment Across TCGA and CGGA

**Figure 7:**
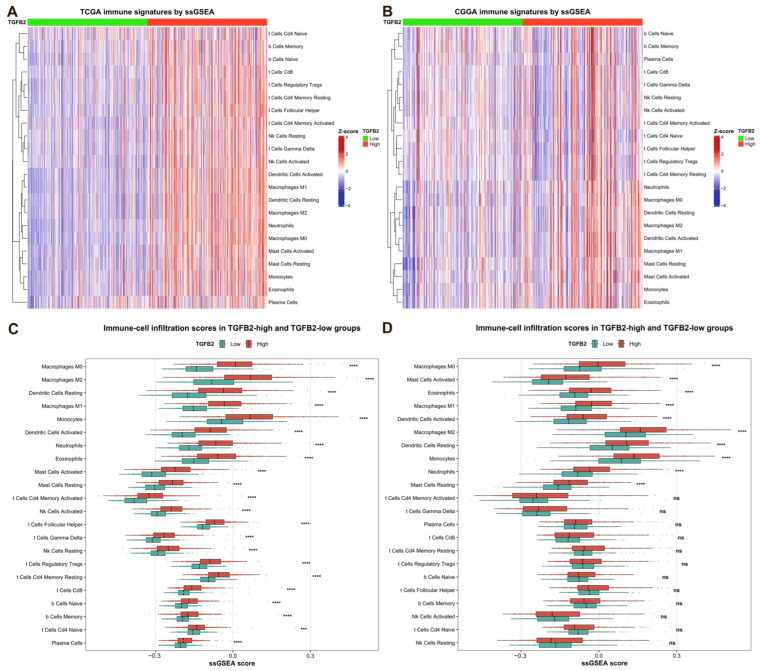
ssGSEA immune-cell signature analysis in TCGA and CGGA glioma cohorts. (**A**,**B**) Row-standardized Z-score heatmaps for 22 immune-cell signatures in the TCGA and CGGA cohorts, respectively. Samples are ordered by TGFB2 expression group. (**C**,**D**) Group-wise comparisons of ssGSEA enrichment scores between TGFB2-high and TGFB2-low tumors in TCGA and CGGA, respectively. Statistical significance was assessed using the Wilcoxon rank-sum test with Benjamini–Hochberg FDR correction. **** *p* < 0.0001; *** *p* < 0.001; ns, not significant.

**Figure 8:**
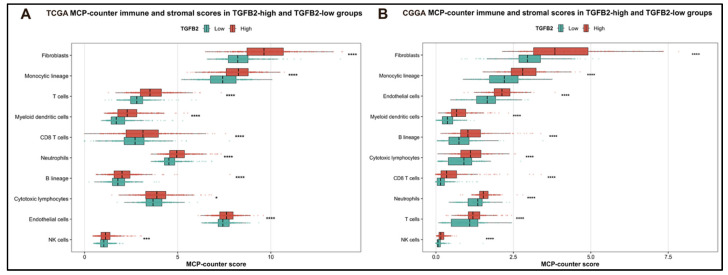
MCP-counter immune and stromal cell abundance scores compared between TGFB2-high and TGFB2-low groups in the TCGA (**A**) and CGGA (**B**) cohorts, respectively. Statistical significance was assessed using the Wilcoxon rank-sum test with Benjamini–Hochberg FDR correction. **** *p* < 0.0001; *** *p* < 0.001; * *p* < 0.05.

In the TCGA discovery cohort, ssGSEA across 22 immune-cell signatures revealed pervasive and statistically significant enrichment in TGFB2-high tumors compared with the TGFB2-low group, as depicted in the heatmap and group-comparison plots (Figure 7A,C). The strongest enrichment shifts involved macrophage (M0, M1, M2), dendritic cell, neutrophil, monocyte, and follicular helper T-cell signatures, with all 22 tested signatures reaching statistical significance after FDR correction (Supplementary Table S3). MCP-counter independently confirmed this pattern, demonstrating significantly higher abundance scores for all 10 immune and stromal populations in TGFB2-high tumors (Figure 8A). The largest absolute differences were observed for fibroblasts (median 9.63 vs. 8.22), monocytic lineage (8.26 vs. 7.42), T cells (3.51 vs. 2.80), and myeloid dendritic cells (2.28 vs. 1.70). Additional significant increases were observed across CD8 T cells, neutrophils, B lineage, cytotoxic lymphocytes, endothelial cells, and natural killer (NK) cells.

In the CGGA validation cohort, the overall direction of effect was preserved but the enrichment pattern was more selective (Figure 7B,D). ssGSEA demonstrated significantly higher enrichment in TGFB2-high tumors for myeloid-associated signatures, including macrophages M0, M1, and M2, activated and resting dendritic cells, monocytes, neutrophils, mast cells, and eosinophils (Supplementary Table S4). By contrast, lymphoid-associated signatures—including CD8 T cells, regulatory T cells, B cells, NK cells, follicular helper T cells, and plasma cells—failed to achieve statistical significance in CGGA, a pattern that distinguishes this cohort from the pan-immune enrichment observed in TCGA. MCP-counter again showed significantly higher abundance scores for all 10 tested immune and stromal populations in TGFB2-high tumors (Figure 8B). The largest differences were observed for fibroblasts (median 3.84 vs. 2.96), monocytic lineage (2.80 vs. 2.21), and endothelial cells (2.13 vs. 1.66), with significant increases also observed across myeloid dendritic cells, B lineage, cytotoxic lymphocytes, CD8 T cells, neutrophils, T cells, and NK cells.

### 3.6. TGFB2 Is Positively Associated with Immune Checkpoint and Immunoregulatory Marker Expression in Both Cohorts

**Figure 9:**
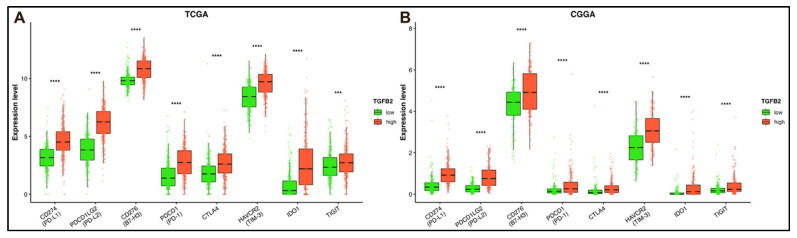
Boxplot comparisons of immune checkpoint gene expression between (PD-L1, TIM-3, *IDO1*, *CTLA4*, *TIGIT*, *ICOS*, and *IL2RA*) TGFB2-high and TGFB2-low groups in the TCGA (**A**) and CGGA (**B**) cohorts, respectively. Statistical significance was assessed using the Wilcoxon rank-sum test. **** *p* < 0.0001; *** *p* < 0.001.

In the TCGA glioma cohort, all tested immune checkpoint genes showed significantly elevated expression in TGFB2-high relative to TGFB2-low tumors. These included *CD274* (PD-L1), *PDCD1LG2* (PD-L2), *CD276* (B7-H3), *PDCD1* (PD-1), *CTLA4*, *HAVCR2* (TIM-3), and *IDO1* (all *p* < 0.0001), as well as *TIGIT* (*p* < 0.001; Figure 9A). The same pattern was independently reproduced in the CGGA validation cohort (Figure 9B). Spearman correlation analysis confirmed significant positive associations between TGFB2 and *CD274* (PD-L1) in both TCGA (ρ = 0.565, *p* = 1.563 × 10^−57^; Supplementary Figure S5A) and CGGA (ρ = 0.671, *p* = 4.006 × 10^−54^; Supplementary Figure S5B), and between TGFB2 and *HAVCR2* (TIM-3) in TCGA (ρ = 0.499, *p* = 2.361 × 10^−43^; Supplementary Figure S5C) and CGGA (ρ = 0.491, *p* = 6.947 × 10^−26^; Supplementary Figure S5D). *CD274* and *HAVCR2* yielded the strongest and most consistent cross-cohort associations, establishing a reproducible relationship between TGFB2 expression and upregulation of major inhibitory checkpoint molecules in glioma (Supplementary Table S5).

**Figure 10:**
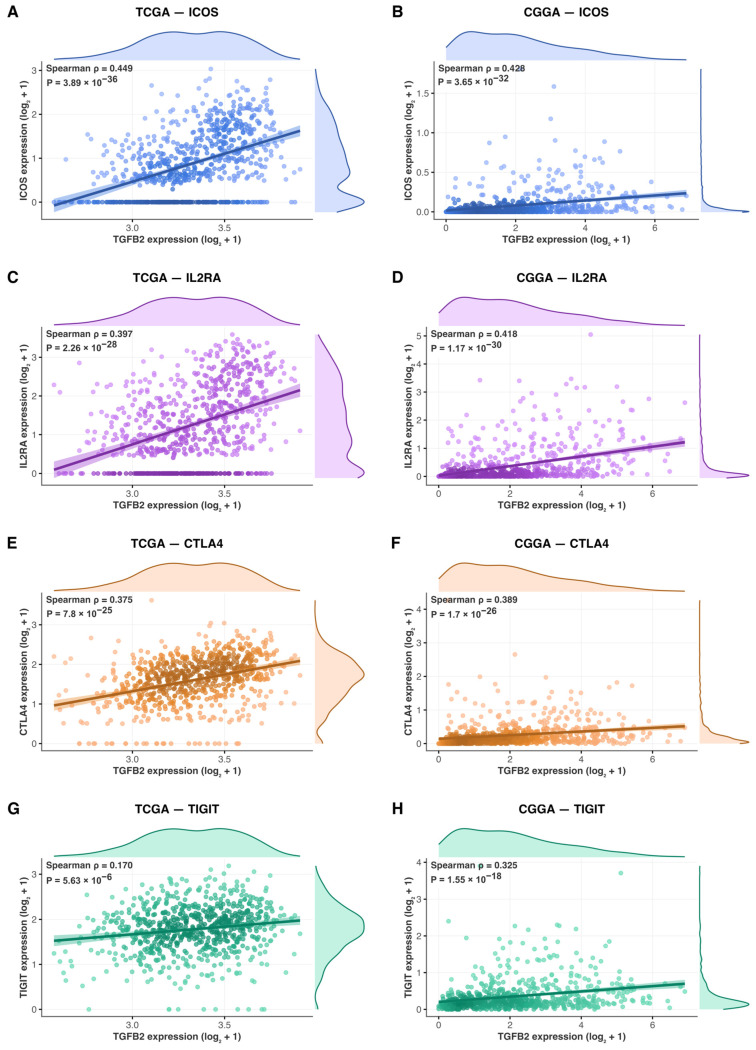
TGFB2 expression is positively correlated with immunoregulatory markers in TCGA and CGGA gliomas. (**A**,**B**) TGFB2 versus *ICOS* in TCGA and CGGA, respectively. (**C**,**D**) TGFB2 versus *IL2RA* in TCGA and CGGA, respectively. (**E**,**F**) TGFB2 versus *CTLA4* in TCGA and CGGA, respectively. (**G**,**H**) TGFB2 versus *TIGIT* in TCGA and CGGA, respectively. Spearman correlation coefficients (ρ) and *p* values are displayed in each panel. Expression values are shown on the log2(x + 1) scale.

To further characterize the immunoregulatory context of TGFB2, Spearman correlation analysis was performed between TGFB2 and four preselected immunoregulatory markers across both cohorts (Figure 10). In TCGA, TGFB2 showed significant positive correlations with *ICOS* (ρ = 0.449, *p* = 3.89 × 10^−36^), *IL2RA* (ρ = 0.397, *p* = 7.26 × 10^−28^), *CTLA4* (ρ = 0.375, *p* = 7.8 × 10^−25^), and *TIGIT* (ρ = 0.170, *p* = 5.63 × 10^−6^; Figure 10A,C,E,G). All four markers were likewise significantly and positively correlated with TGFB2 in CGGA: *ICOS* (ρ = 0.428, *p* = 3.65 × 10^−32^), *IL2RA* (ρ = 0.418, *p* = 1.17 × 10^−30^), *CTLA4* (ρ = 0.389, *p* = 1.7 × 10^−26^), and *TIGIT* (ρ = 0.325, *p* = 1.55 × 10^−18^; Figure 10B,D,F,H). The strongest and most consistent cross-cohort associations were observed for *ICOS*, IL2RA, and *CTLA4*. *TIGIT* showed a reproducible but notably weaker correlation in TCGA (ρ = 0.170) compared with CGGA (ρ = 0.325), a nearly twofold difference in effect size that may reflect differences in cohort composition and IDH distribution rather than a true biological inconsistency; accordingly, *TIGIT* is considered a secondary finding relative to the other three markers. *FOXP3* was additionally screened but showed inconsistent associations across cohorts and was not included in the primary analysis.

### 3.7. Single-Cell Analysis Localizes TGFB2 to Malignant and Myeloid Compartments in Glioma

**Figure 11:**
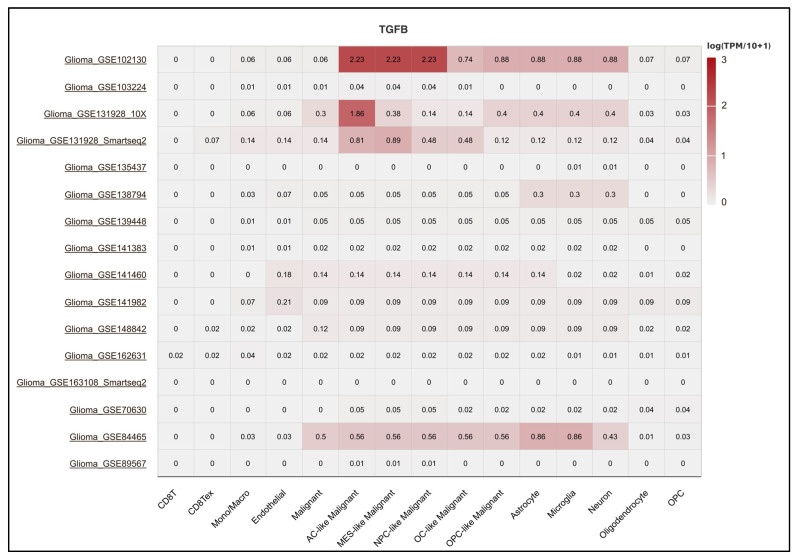
Single-cell localization of TGFB2 across GBM microenvironments based on TISCH2. Heatmap showing average TGFB2 expression across 15 annotated cell-type populations in 16 GBM single-cell RNA-seq datasets from TISCH2. TGFB2 expression is consistently enriched in malignant and myeloid/monocyte–macrophage-related populations across datasets, whereas T-cell populations show low or negligible expression. Color scale represents log(TPM/10 + 1).

To investigate the cellular sources underlying the myeloid-enriched microenvironment observed in the bulk cohort analyses, single-cell RNA-seq datasets were queried through TISCH2. Screening across all 17 available GBM single-cell datasets in TISCH2, of which 16 showed detectable TGFB2 expression and were retained in the heatmap visualization, demonstrated that TGFB2 expression was preferentially enriched in malignant cell populations and myeloid/monocyte–macrophage-related compartments, while T-cell lineages, including CD8 T cells, CD4 conventional T cells, and proliferating T cells, exhibited consistently low to negligible TGFB2 expression across datasets (Figure 11). As GBM-annotated datasets represent the primary glioma tumor type available with standardized cell-type annotation in TISCH2, the cellular localization findings reported here predominantly reflect the GBM microenvironment and should be interpreted with caution when extrapolated to the IDH-mutant LGG cases that constitute the majority of the TCGA and CGGA cohorts analyzed above.

Detailed analysis of two representative datasets confirmed and extended this pattern (Supplementary Figure S6). In GSE148842, TGFB2 expression was detectable primarily in monocyte/macrophage (Mono/Macro) clusters and to a lesser degree in malignant cell populations, while CD8 exhausted T cells and oligodendrocytes showed near-absent expression. In GSE84465, a dataset with broader lineage representation including astrocytes, neurons, oligodendrocyte precursor cells, and vascular cells, the strongest TGFB2 signal was observed in astrocyte-like (AC-like) malignant populations, with moderate expression in Mono/Macro and vascular compartments, and low expression in oligodendrocyte precursor cell (OPC) and oligodendrocyte clusters. Across both datasets, lymphoid populations were consistently among the lowest TGFB2-expressing cell types.

## 4. Discussion

The near-universal failure of immune checkpoint inhibitor monotherapy in glioblastoma underscores that the glioma immune microenvironment is not merely immunologically cold, but is actively suppressed via multiple distinct mechanisms in ways that single-axis blockade cannot overcome [59,60]. This directly reflects the translational gap noted at the outset of this study, wherein the broader success of immune checkpoint blockade across solid tumors has not yet been matched by comparable benefit in glioblastoma [13]. The present multi-cohort transcriptomic analysis indicates that TGFB2 is a consistent molecular correlate of this suppressive state across two independent glioma cohorts. TGFB2-high tumors are characterized by a myeloid- and stromal-enriched microenvironment, concordant upregulation of multiple immune-checkpoint and immunoregulatory axes including PD-L1, TIM-3, *IDO1*, *CTLA4*, and *TIGIT*, and a transcriptional program dominated by inflammatory, mesenchymal, and stress-adaptive signaling. That this expression pattern also predicts worse overall survival, and retains independent prognostic significance in the CGGA validation cohort after full molecular adjustment, positions TGFB2 as both an immunologically and prognostically informative marker that complements existing WHO CNS5 classifiers [2]. This dual informativeness reflects the broader value of prognostic biomarkers in precision oncology, where molecules capable of simultaneously informing risk stratification and immunological subtyping are particularly well suited to guide patient-specific therapeutic decision-making [26].

Before interpreting these associations mechanistically, several analytical constraints merit explicit acknowledgement. This is a transcriptomic biomarker study, and the observed relationships between TGFB2 and immune or transcriptional variables are correlational; they do not establish whether TGFB2 mechanistically drives the identified microenvironmental states or whether it is itself a transcriptional readout of a broader tumor program.

Immune enrichment scores derived by ssGSEA were calculated from the same expression matrices used to define TGFB2-high and TGFB2-low groups; although ssGSEA signatures do not include TGFB2 itself, co-regulated inflammatory programs in glioma may partially inflate enrichment estimates, and MCP-counter, which uses a curated, non-overlapping gene set, is therefore considered the primary immune quantification result. More broadly, these computational deconvolution outputs represent inferred relative cell-type abundance rather than direct cellular measurements such as flow cytometry, immunohistochemistry, or single-cell quantification, and should be interpreted accordingly. Because bulk RNA-seq immune deconvolution methods including both ssGSEA and MCP-counter cannot distinguish resident microglia from peripherally recruited monocyte-derived macrophages, the myeloid signal reported here integrates signals from both compartments, a distinction that is biologically and clinically important in the glioma context and that could not be resolved from these data alone. Molecular annotation for IDH, MGMT, and 1p/19q in TCGA required supplementation from cBioPortal, introducing potential heterogeneity. The mechanistic questions raised by these correlational findings, including whether TGF-β2 isoform specifically drives myeloid polarization and checkpoint upregulation, and whether TGFB2 expression predicts response to combined TGF-β/checkpoint blockade, are the subject of a prospective experimental program.

The malignancy-ordered expression gradient confirmed across TCGA and CGGA, and corroborated at the protein level in GBM through UALCAN/CPTAC proteomic data, is consistent with prior experimental evidence identifying TGF-β2 as the principal TGF-β isoform produced by high-grade glioma cells [18,61]. The protein-level CPTAC data, while exploratory given the limited normal tissue sample size (*n* = 18) and the methodological differences between mass spectrometry proteomics and RNA-seq quantification, provide supporting evidence that this expression gradient is not restricted to the transcriptomic level. Its enrichment in IDH-wildtype, non-codeleted, and MGMT-unmethylated tumors, the molecular fingerprint of the most clinically aggressive glioma subtype under the 2021 WHO CNS5 classification [2], suggests that TGFB2 expression co-segregates with the broader transcriptional state of molecularly aggressive glioma, rather than representing an isolated signaling event. Recent studies have similarly reported that TGFB2 mRNA levels are adverse prognostic markers in low-grade glioma and that TGFB2 methylation status significantly impacts GBM survival, further supporting its relevance as a clinically informative marker across the glioma spectrum [62,63].

The attenuation of TGFB2-independent prognostic significance in the TCGA multivariate model, where IDH status, WHO grade, and age collectively account for the majority of explained variance, reflects a well-recognized statistical consequence for transcriptomic markers that are biologically downstream of, and therefore partially mediated through, the dominant molecular classifiers in glioma. Prognostic mediation of this kind differs conceptually from confounding: whereas confounding implies that a marker–outcome association is explained by an unrelated covariate, mediation implies that the marker operates through a causal pathway shared with the covariate, such that adjustment attenuates rather than eliminates the underlying biological relationship. TGFB2 upregulation is a transcriptional feature associated with the IDH-wildtype mesenchymal state, and its univariate prognostic signal is therefore partially captured by IDH status and grade in a fully adjusted model. The retention of independent significance in the CGGA multivariate model (HR = 1.343, 95% CI 1.144–1.577; *p* = 3.2 × 10^−4^) after full adjustment for the same covariates provides the more meaningful cross-validated estimate. The asymmetry between cohorts likely reflects differences in cohort composition and expression dynamic range rather than a true biological discrepancy, and the convergence of univariate findings across both cohorts supports a reproducible adverse prognostic association. The non-significance of MGMT promoter methylation in both multivariate models, TCGA (*p* = 0.174) and CGGA (*p* = 0.941), is consistent with the well-documented attenuation of MGMT methylation prognostic effect when IDH mutation status is included as a covariate, as the two variables are strongly co-correlated and IDH status captures a broader share of the explained survival variance in molecularly mixed glioma cohorts [64].

The non-monotonic grade distribution observed in CGGA, where grade III tumors showed marginally lower median TGFB2 expression than grade II, likely reflects the heterogeneous molecular composition of WHO grade III gliomas in this cohort, which includes both IDH-mutant astrocytomas and oligodendrogliomas with divergent TGFB2 expression levels, rather than representing a true biological departure from the malignancy-associated expression gradient.

The transcriptional state of TGFB2-high gliomas is broader than canonical TGF-β pathway activation alone. The strongest Hallmark enrichment in TCGA was interferon-γ response (NES = 3.220), followed by epithelial–mesenchymal transition (NES = 2.867), TNF-α/NF-κB signaling (NES = 2.692), and inflammatory response (NES = 2.604). The co-dominance of interferon and TGF-β-associated signatures in the same tumor group requires interpretive care. One possible interpretation is that TGFB2-high status is simply a correlate of overall immune reactivity, and that the interferon signal reflects genuine and effective anti-tumor immunity. However, this interpretation is inconsistent with the concurrent findings of the present study: if interferon-γ signaling were driving effective immune clearance, one would not expect the simultaneous co-elevation of PD-L1, TIM-3, *IDO1*, *CTLA4*, and *TIGIT* observed in TGFB2-high tumors across both cohorts. The more parsimonious interpretation, supported by the adaptive immune resistance framework, is that interferon-γ exposure in TGFB2-high tumors induces checkpoint ligand upregulation, particularly PD-L1 via JAK/STAT signaling, while TGF-β2 simultaneously suppresses effector T-cell function, generating a state of immune activation without productive immune clearance [65,66]. This interpretation is further supported by Trieu et al. [63], who specifically identified co-expression of TGFB2 with interferon regulatory factor signaling in glioma as a feature of immunosuppressive rather than immunoprotective biology. The co-enrichment of EMT, angiogenesis, hypoxia, and mTORC1 signaling hallmarks is consistent with a mesenchymal-like transcriptional state associated with high immune infiltration, poor prognosis, and therapy resistance in glioblastoma [67,68,69]. TGF-β is a canonical driver of EMT in glioma, promoting invasion through SMAD-dependent induction of mesenchymal transcription factors including SLUG and ZEB1 [19], and RUNX2-mediated TGF-β/SMAD signaling has been linked to malignant progression in IDH-wildtype tumors [21].

The immune analyses consistently identified myeloid and stromal populations as the most reproducible cross-cohort correlates of TGFB2-high expression. Both ssGSEA and MCP-counter demonstrated significantly higher fibroblast, monocytic lineage, dendritic cell, and macrophage-associated scores in TGFB2-high tumors across TCGA and CGGA, with the myeloid and stromal components representing the strongest and most directionally consistent signals. This pattern is concordant with TGF-β2’s established role in polarizing glioma-associated myeloid cells toward immunosuppressive phenotypes through SMAD-dependent and SMAD-independent mechanisms [18,70]. In the glioma context, this myeloid compartment encompasses two biologically distinct populations that bulk deconvolution cannot separate: resident microglia and peripherally recruited monocyte-derived macrophages (MDMs) [71,72]. These populations differ substantially in their developmental origin, transcriptional identity, TGF-β responsiveness, and functional contributions to the immunosuppressive niche. TGF-β2 has been shown experimentally to suppress microglial antigen-presenting capacity and promote a homeostatic-to-suppressive phenotypic transition through SMAD3-dependent mechanisms [71], distinct from its effects on peripheral MDMs. The clinical importance of resolving this distinction has been further underscored by recent single-cell analyses demonstrating that distinct myeloid-derived suppressor cell populations in human GBM exert non-redundant immunosuppressive functions with differential therapeutic vulnerabilities [73]. The predominantly myeloid ssGSEA enrichment pattern observed in CGGA, where lymphoid signatures were selectively non-significant while myeloid signals remained robust, is consistent with a microglial and monocyte/macrophage-dominated suppressive niche. Spatially resolved multi-omics analyses have further demonstrated that bidirectional tumor–myeloid interactions reinforce mesenchymal cellular states and the immunosuppressive niche in glioblastoma [74]. The TISCH2 single-cell evidence that TGFB2 localizes predominantly to malignant and monocyte/macrophage populations is consistent with bulk TGFB2 expression integrating signals from both neoplastic and innate immune compartments, offering a plausible cellular basis for the marker’s association with both malignant phenotype and myeloid-enriched immunosuppressive biology. Future work combining spatially resolved or single-cell approaches with immunohistochemistry or multiplex immunofluorescence in an independent surgical glioma cohort would both resolve the relative contributions of resident microglia versus MDMs and confirm TGFB2 protein-level co-localization with malignant and myeloid cell populations in situ. Glioma-associated myeloid cells constitute up to 30–50% of the GBM tumor mass and represent a primary barrier to effective immunotherapy in this disease [72]; the consistent association of TGFB2 with this compartment therefore has direct implications for understanding the immune-evasive architecture of TGFB2-high gliomas [75].

The convergent checkpoint co-expression findings further support the immunosuppressive microenvironmental interpretation. TGFB2 expression was significantly and positively correlated with *CD274*/PD-L1 (ρ = 0.565 TCGA, ρ = 0.671 CGGA), *HAVCR2*/TIM-3 (ρ = 0.499 TCGA, ρ = 0.491 CGGA), *IDO1*, *PDCD1LG2*/PD-L2, *ICOS*, *IL2RA*, *CTLA4*, and *TIGIT* across both cohorts. The co-elevation of PD-L2 alongside PD-L1 is of clinical interest because PD-L2 independently suppresses T-cell activation and has been associated with resistance to anti-PD-1 therapy in tumors with high PD-L1 expression [76]. The positive associations with *ICOS* and *IL2RA*, markers linked to regulatory T-cell activity and T-cell activation thresholds, are consistent with a microenvironment in which co-stimulatory and co-inhibitory axes are simultaneously engaged, potentially reflecting a Treg-associated immunoregulatory state rather than a productive cytotoxic response. The TIM-3 and IDO1 associations suggest that tryptophan-catabolism-driven metabolic immunosuppression may co-exist with checkpoint-mediated suppression in TGFB2-high gliomas [77], though this interpretation is inferential given the transcriptomic nature of the present data. This convergent pattern of multi-axis immunosuppressive co-expression offers a framework for understanding the repeated failure of single-agent checkpoint blockade in glioblastoma: when PD-L1, TIM-3, *IDO1*, and TGF-β axes are simultaneously co-expressed, inhibition of any single axis is unlikely to be sufficient [59,60]. These findings are consistent with TGFB2 functioning as the type of upstream coordinating node proposed in current combination-immunotherapy frameworks, in which a single regulatory factor is associated with simultaneous engagement of multiple, mechanistically distinct immune checkpoint pathways [20].

The clinical experience with TGF-β inhibition in glioma has been uniformly disappointing: galunisertib failed to improve overall survival in both newly diagnosed and recurrent GBM [22,23], and trabedersen was terminated for futility at Phase III [24]. The multi-axis immunosuppressive portrait described here, with simultaneous co-expression of PD-L1, TIM-3, IDO1, and TGF-β-associated transcriptional programs, is consistent with the mechanistic explanation that single-agent TGF-β blockade is insufficient in tumors where multiple redundant immunosuppressive axes are co-expressed, and that adaptive upregulation of compensatory pathways likely limits monotherapy efficacy [25]. These observations support the biological plausibility of combination approaches, including bifunctional TGF-β/PD-L1 inhibitors such as bintrafusp alfa [78] and TGFβRII-modified CAR-T cell strategies [79]; however, the broader clinical experience with combined TGF-β/checkpoint blockade across tumor types has highlighted that the absence of biomarker-driven patient selection is a primary driver of treatment failure [80], further underscoring the translational rationale for TGFB2 expression-based stratification proposed here. Whether TGFB2 expression can serve as a patient-selection biomarker in such trials will be addressed through the prospective experimental program described above, which is designed to provide the mechanistic and preclinical basis for TGFB2-stratified patient selection in combination immunotherapy trials.

## 5. Conclusions

Taken together, the present findings position TGFB2 as a transcriptomic marker that bridges two critical and interconnected features of aggressive glioma: a myeloid- and stromal-enriched tumor microenvironment driven by innate immune compartments that bulk deconvolution identifies as predominantly monocytic and macrophage-associated, and a convergent multi-axis immunosuppressive state in which PD-L1, TIM-3, *IDO1*, *CTLA4*, *TIGIT*, *ICOS*, and *IL2RA* are simultaneously co-expressed. These findings are reinforced by the consistent overexpression of TGFB2 across both cohorts, its robust association with adverse survival outcomes (3-year AUC of 0.823 in TCGA and 0.714 in CGGA), and the concordant enrichment of immunosuppressive checkpoint and innate immune signatures, collectively supporting TGFB2 as a clinically relevant biomarker of immune dysfunction in diffuse glioma. By integrating prognostic and immunological dimensions within a single transcriptomic marker, this study exemplifies the type of multi-functional biomarker increasingly sought in precision oncology. This co-activation pattern, reproduced across two independent cohorts and localized at the single-cell level to malignant and myeloid compartments, offers a plausible molecular basis for understanding why the glioma immune microenvironment resists single-agent therapeutic intervention, and why TGFB2-high tumors in particular may represent a subgroup in which redundant immunosuppressive pathway engagement is especially pronounced. The present transcriptomic findings define three mechanistic questions that will be addressed in a prospective experimental program. First, whether TGF-β2 isoform-specifically polarizes human microglia toward an immunosuppressive phenotype through canonical SMAD3 signaling, a gap left unresolved by prior studies using pan-TGF-β inhibition or TGF-β1 specifically, will be tested using recombinant TGF-β2 stimulation in human microglial models with selective SMAD3 inhibition and functional co-culture readouts including CD8^+^ T-cell cytotoxic assays. Second, whether tumor-derived TGFB2 directly drives checkpoint upregulation in glioma and myeloid compartments, and whether this proceeds through canonical SMAD or compensatory interferon/JAK/STAT-mediated mechanisms, a mechanistic distinction of direct clinical relevance given the failure of pan-TGF-β blockade in GBM, will be addressed through TGFB2 knockdown in IDH-wildtype glioma cell lines and conditioned medium transfer to microglia, with checkpoint expression and signaling pathway interrogation as primary endpoints. Third, whether TGFB2 expression level predicts sensitivity to combined TGF-β/checkpoint blockade will be evaluated in the GL261 syngeneic orthotopic model, providing proof-of-concept for TGFB2 as a patient-stratification variable in trials of bifunctional agents such as bintrafusp alfa. Together, these experimental objectives are designed to causally link the transcriptomic associations reported here to specific immunosuppressive mechanisms and therapeutic vulnerabilities; should they confirm the proposed mechanistic links, TGFB2 may serve not only as a prognostic indicator but also as a clinically actionable stratification biomarker for selecting glioma patients most likely to benefit from combination TGF-β/checkpoint blockade immunotherapy.

## Figures and Tables

**Table 1:** Clinicopathological characteristics of the TCGA and CGGA glioma cohorts.

Characteristic	TCGA (*n* = 667)	CGGA (*n* = 404)
**Age (years) ^1^**		
≤40	266	164
>40	401	239
**Sex**		
Male	384	231
Female	283	173
**WHO Grade ^2^**		
II	248	130
III	265	141
IV	153	133
**Histological subtype**		
Astrocytoma	194	158
Oligodendroglioma	190	90
Oligoastrocytoma	130	23
Glioblastoma	153	133
**IDH status ^3^**		
Mutant	423	197
Wild-type	244	170
**1p/19q codeletion status ^4^**		
Codeletion	172	85
Non-codeletion	484	262
**MGMT promoter methylation ^5^**		
Methylated	299	190
Unmethylated	338	143
**WHO CNS5 molecular class ^6^**		
Astrocytoma, IDH-mutant, 1p/19q non-codeleted	251	106
Oligodendroglioma, IDH-mutant, 1p/19q codeleted	170	66
Glioblastoma, IDH-wild-type	144	105

^1^ CGGA: 1 case missing age. ^2^ TCGA: 1 case missing grade. ^3^ CGGA: 37 cases missing IDH status. ^4^ 11 TCGA and 57 CGGA cases with unavailable 1p/19q annotation excluded from codeletion analysis. ^5^ 30 TCGA and 71 CGGA cases excluded from MGMT methylation analysis. ^6^ WHO CNS5 class assigned only to molecularly complete cases. Oligoastrocytoma (TCGA *n* = 130; CGGA *n* = 23) is not recognized under WHO CNS5 and was excluded from molecular class assignment; these cases were retained for all other analyses and reclassified where complete molecular annotation permitted.

**Table 2:** Prognostic performance of TGFB2 expression in the TCGA and CGGA glioma cohorts.

Cohort	n	Cutoff	Low n	High n	Median OS Low (Days)	Median OS High (Days)	Log-Rank *p*	1-Year AUC	2-Year AUC	3-Year AUC
TCGA	664	Median TGFB2 = 8.974	332	332	2907	648	<0.0001	0.761	0.800	0.823
CGGA	404	Median TGFB2 = 2.245	202	202	3107	863	<0.0001	0.621	0.710	0.714

**Table 3:** Univariate and multivariate Cox proportional hazards regression analyses of overall survival in the TCGA glioma cohort.

Variable	Univariate HR (95% CI)	*p* (Univariate)	Multivariate HR (95% CI)	*p* (Multivariate)
TGFB2 expression	1.474 (1.368–1.589)	2.4 × 10^−24^	1.049 (0.938–1.173)	0.402
WHO grade III	3.295 (2.227–4.874)	2.4 × 10^−9^	2.262 (1.509–3.391)	7.8 × 10^−5^
WHO grade IV	21.896 (14.264–33.612)	3.1 × 10^−45^	4.801 (2.894–7.965)	1.2 × 10^−9^
Age	1.069 (1.058–1.080)	1.5 × 10^−37^	1.039 (1.027–1.052)	4.5 × 10^−10^
IDH mutation	0.116 (0.087–0.155)	9.8 × 10^−48^	0.407 (0.261–0.636)	7.6 × 10^−5^
MGMT methylated	0.716 (0.538–0.954)	0.023	0.810 (0.598–1.098)	0.174
1p/19q codeletion	0.281 (0.185–0.427)	2.5 × 10^−9^	0.677 (0.404–1.136)	0.139

**Table 4:** Univariate and multivariate Cox proportional hazards regression analyses of overall survival in the CGGA glioma cohort.

Variable	Univariate HR (95% CI)	*p* (Univariate)	Multivariate HR (95% CI)	*p* (Multivariate)
TGFB2 expression	1.821 (1.614–2.055)	2.5 × 10^−22^	1.343 (1.144–1.577)	3.2 × 10^−4^
WHO grade III	2.650 (1.517–4.628)	6.2 × 10^−4^	2.753 (1.574–4.815)	3.8 × 10^−4^
WHO grade IV	10.074 (5.883–17.248)	3.8 × 10^−17^	3.685 (1.982–6.850)	3.7 × 10^−5^
Age	1.036 (1.022–1.050)	2.7 × 10^−7^	1.013 (1.000–1.026)	0.049
IDH mutation	0.226 (0.159–0.320)	9.0 × 10^−17^	0.601 (0.380–0.949)	0.029
MGMT methylated	0.724 (0.521–1.007)	0.055	0.987 (0.699–1.395)	0.941
1p/19q codeletion	0.167 (0.088–0.318)	5.5 × 10^−8^	0.393 (0.195–0.794)	0.009

Analysis based on complete cases. HR, hazard ratio; CI, confidence interval.

## Data Availability

The raw data supporting the findings of this study are publicly available from the following repositories: TCGA RNA-seq expression and clinical data were downloaded from the Genomic Data Commons (GDC) portal (https://gdc.cancer.gov/). Molecular annotation for TCGA samples was obtained from cBioPortal PanCancer Atlas (https://www.cbioportal.org/). CGGA RNA-seq (mRNAseq_693) expression and clinical data were downloaded from the CGGA portal (https://www.cgga.org.cn/). Single-cell RNA-seq data were accessed through TISCH2 (https://tisch.compbio.cn/). Protein-level expression data were accessed through UALCAN (https://ualcan.path.uab.edu). Processed results supporting all figures and conclusions are provided as Supplementary Tables S1–S5 and Supplementary Figures S1–S6. Additional data tables and all R analysis scripts are available from the corresponding author upon reasonable request.

## Supplementary Materials

The following supporting information can be downloaded at: https://www.mdpi.com/article/10.3390/cancers18132092/s1, Table S1: TCGA Hallmark GSEA full results. Full Hallmark GSEA results for TGFB2-high vs TGFB2-low in the TCGA glioma cohort. Positive and negative enrichment directions shown. Ranked by adjusted *p* value within each direction; Table S2: CGGA Hallmark GSEA full results. Full Hallmark GSEA results for TGFB2-high vs. TGFB2-low in the CGGA glioma cohort. Positive enrichment direction only. Ranked by adjusted *p* value; Table S3: TCGA ssGSEA immune-cell signature comparison. ssGSEA immune-cell signature comparison between TGFB2-high and TGFB2-low groups in the TCGA glioma cohort (n = 667). All 22 signatures reached statistical significance after FDR correction; Table S4: CGGA ssGSEA immune-cell signature comparison. ssGSEA immune-cell signature comparison between TGFB2-high and TGFB2-low groups in the CGGA glioma cohort (n = 404). Myeloid signatures reached significance; lymphoid signatures were non-significant; Table S5: Immune checkpoint Spearman correlations in TCGA and CGGA. Spearman correlation between TGFB2 and immune checkpoint gene expression in TCGA (n = 667) and CGGA (n = 404) glioma cohorts; Figure S1: Pan-cancer CPTAC proteomic profiling of TGFB2; Figure S2: TGFB2 expression across clinicopathological subgroups in TCGA and CGGA; Figure S3: GO enrichment analysis of upregulated genes in TGFB2-high tumors in TCGA and CGGA; Figure S4: KEGG pathway enrichment analysis of upregulated genes in TGFB2-high tumors in TCGA and CGGA; Figure S5: Spearman correlation between TGFB2 and *CD274* (PD-L1) and *HAVCR2* (TIM-3) in TCGA and CGGA; Figure S6: Single-cell TGFB2 expression in representative GBM datasets from TISCH2.

## Abbreviations

The following abbreviations are used in this manuscript:

AUC	Area under the curve
CGGA	Chinese Glioma Genome Atlas
CPTAC	Clinical Proteomic Tumor Analysis Consortium
DEGs	Differentially expressed genes
FDR	False discovery rate
FPKM	Fragments Per Kilobase Million
GAMs	Glioma-associated macrophages and microglia
GDC	Genomic Data Commons
GSEA	Gene set enrichment analysis
IDO1	Indoleamine 2,3-dioxygenase 1
IL2RA	Interleukin-2 receptor subunit alpha
ICOS	Inducible T-cell co-stimulator
MCP-counter	Microenvironment Cell Populations counter
mTORC1	Mechanistic target of rapamycin complex 1
NES	Normalized enrichment score
OPC	Oligodendrocyte precursor cell
ssGSEA	Single-sample gene set enrichment analysis
TCGA	The Cancer Genome Atlas
TIGIT	T-cell immunoreceptor with Ig and ITIM domains
TISCH2	Tumor Immune Single-cell Hub 2

## References

[1] Weller M., van den Bent M., Preusser M., Le Rhun E., Tonn J.C., Minniti G., Bendszus M., Balana C., Chinot O., Dirven L. (2021). EANO Guidelines on the Diagnosis and Treatment of Diffuse Gliomas of Adulthood. Nat. Rev. Clin. Oncol..

[2] Louis D.N., Perry A., Wesseling P., Brat D.J., Cree I.A., Figarella-Branger D., Hawkins C., Ng H.K., Pfister S.M., Reifenberger G. (2021). The 2021 WHO Classification of Tumors of the Central Nervous System: A Summary. Neuro Oncol..

[3] Bray F., Laversanne M., Sung H., Ferlay J., Siegel R.L., Soerjomataram I., Jemal A. (2024). Global Cancer Statistics 2022: GLOBOCAN Estimates of Incidence and Mortality Worldwide for 36 Cancers in 185 Countries. CA Cancer J. Clin..

[4] Price M., Ballard C.A.P., Benedetti J.R., Kruchko C., Barnholtz-Sloan J.S., Ostrom Q.T. (2025). CBTRUS Statistical Report: Primary Brain and Other Central Nervous System Tumors Diagnosed in the United States in 2018–2022. Neuro-Oncol..

[5] Jiang T., Tang G., Lin Y., Peng X., Zhang X., Zhai X., Peng X., Yang J., Huang H., Wu N. (2011). Prevalence Estimates for Primary Brain Tumors in China: A Multi-Center Cross-Sectional Study. Chin. Med. J..

[6] Tu S., Huang X., Fan X., Zhang Y., Huang L., Li K., Wang X., Gao Y., Zhao X., Feng Z. (2025). National and Subnational Burden of Brain and Central Nervous System Cancers in China and Global from 1990 to 2021: Results from the Global Burden of Disease Study 2021. Arch. Public Health.

[7] Xiao D., Yan C., Li D., Xi T., Liu X., Zhu D., Huang G., Xu J., He Z., Wu A. (2023). National Brain Tumour Registry of China (NBTRC) Statistical Report of Primary Brain Tumours Diagnosed in China in Years 2019–2020. Lancet Reg. Health West. Pac..

[8] Lerner A., Palmer K., Campion T., Millner T.O., Scott E., Lorimer C., Paraskevopoulos D., McKenna G., Marino S., Lewis R. (2024). Gliomas in Adults: Guidance on Investigations, Diagnosis, Treatment and Surveillance. Clin. Med..

[9] Mo Z., Xin J., Chai R., Woo P.Y.M., Chan D.T.M., Wang J. (2022). Epidemiological Characteristics and Genetic Alterations in Adult Diffuse Glioma in East Asian Populations. Cancer Biol. Med..

[10] Wei J., Li Y., Zhou W., Ma X., Hao J., Wen T., Li B., Jin T., Hu M. (2024). The Construction of a Novel Prognostic Prediction Model for Glioma Based on GWAS-Identified Prognostic-Related Risk Loci. Open Med..

[11] Stupp R., Mason W.P., van den Bent M.J., Weller M., Fisher B., Taphoorn M.J.B., Belanger K., Brandes A.A., Marosi C., Bogdahn U. (2005). Radiotherapy plus Concomitant and Adjuvant Temozolomide for Glioblastoma. N. Engl. J. Med..

[12] Tykocki T., Eltayeb M. (2018). Ten-Year Survival in Glioblastoma. A Systematic Review. J. Clin. Neurosci..

[13] Badani A., Ozair A., Khasraw M., Woodworth G.F., Tiwari P., Ahluwalia M.S., Mansouri A. (2025). Immune Checkpoint Inhibitors for Glioblastoma: Emerging Science, Clinical Advances, and Future Directions. J. Neurooncol..

[14] Sahm F., Brandner S., Bertero L., Capper D., French P.J., Figarella-Branger D., Giangaspero F., Haberler C., Hegi M.E., Kristensen B.W. (2023). Molecular Diagnostic Tools for the World Health Organization (WHO) 2021 Classification of Gliomas, Glioneuronal and Neuronal Tumors; an EANO Guideline. Neuro-Oncol..

[15] Derynck R., Turley S.J., Akhurst R.J. (2021). TGFβ Biology in Cancer Progression and Immunotherapy. Nat. Rev. Clin. Oncol..

[16] Deng Z., Fan T., Xiao C., Tian H., Zheng Y., Li C., He J. (2024). TGF-β Signaling in Health, Disease and Therapeutics. Signal Transduct. Target. Ther..

[17] Batlle E., Massagué J. (2019). Transforming Growth Factor-β Signaling in Immunity and Cancer. Immunity.

[18] Hau P., Jachimczak P., Schlaier J., Bogdahn U. (2011). TGF-Β2 Signaling in High-Grade Gliomas. Curr. Pharm. Biotechnol..

[19] Han J., Alvarez-Breckenridge C.A., Wang Q.-E., Yu J. (2015). TGF-β Signaling and Its Targeting for Glioma Treatment. Am. J. Cancer Res..

[20] Liu Y., Yadav U.P., Chen C., Wang P. (2026). Dual Immune Checkpoint Blockade Strategies for Cancer Therapy. Cell Investig..

[21] Zhou P., Wei Q., Wang H., Yu T., Chen L., Huang Q., Wei L., Jiang J. (2025). RUNX2 Mediated Epithelial Mesenchymal Transition Induced by TGF Beta and Smad Signaling Promotes Malignant Progression in Glioma. Sci. Rep..

[22] Brandes A.A., Carpentier A.F., Kesari S., Sepulveda-Sanchez J.M., Wheeler H.R., Chinot O., Cher L., Steinbach J.P., Capper D., Specenier P. (2016). A Phase II Randomized Study of Galunisertib Monotherapy or Galunisertib plus Lomustine Compared with Lomustine Monotherapy in Patients with Recurrent Glioblastoma. Neuro-Oncol..

[23] Wick A., Desjardins A., Suarez C., Forsyth P., Gueorguieva I., Burkholder T., Cleverly A.L., Estrem S.T., Wang S., Lahn M.M. (2020). Phase 1b/2a Study of Galunisertib, a Small Molecule Inhibitor of Transforming Growth Factor-Beta Receptor I, in Combination with Standard Temozolomide-Based Radiochemotherapy in Patients with Newly Diagnosed Malignant Glioma. Investig. New Drugs.

[24] Bogdahn U., Hau P., Stockhammer G., Venkataramana N.K., Mahapatra A.K., Suri A., Balasubramaniam A., Nair S., Oliushine V., Parfenov V. (2011). Targeted Therapy for High-Grade Glioma with the TGF-Β2 Inhibitor Trabedersen: Results of a Randomized and Controlled Phase IIb Study. Neuro-Oncol..

[25] Capper D., von Deimling A., Brandes A.A., Carpentier A.F., Kesari S., Sepulveda-Sanchez J.M., Wheeler H.R., Chinot O., Cher L., Steinbach J.P. (2017). Biomarker and Histopathology Evaluation of Patients with Recurrent Glioblastoma Treated with Galunisertib, Lomustine, or the Combination of Galunisertib and Lomustine. Int. J. Mol. Sci..

[26] Becatti M., Zheng S., Bronte G. (2025). Editorial: Cancer Biomarkers: Molecular Insights into Diagnosis, Prognosis, and Risk Prediction. Front. Mol. Biosci..

[27] Weinstein J.N., Collisson E.A., Mills G.B., Shaw K.R.M., Ozenberger B.A., Ellrott K., Shmulevich I., Sander C., Stuart J.M., The Cancer Genome Atlas Research Network (2013). The Cancer Genome Atlas Pan-Cancer Analysis Project. Nat. Genet..

[28] Zhao Z., Zhang K.-N., Wang Q., Li G., Zeng F., Zhang Y., Wu F., Chai R., Wang Z., Zhang C. (2021). Chinese Glioma Genome Atlas (CGGA): A Comprehensive Resource with Functional Genomic Data from Chinese Glioma Patients. Genom. Proteom. Bioinform..

[29] Han Y., Wang Y., Dong X., Sun D., Liu Z., Yue J., Wang H., Li T., Wang C. (2023). TISCH2: Expanded Datasets and New Tools for Single-Cell Transcriptome Analyses of the Tumor Microenvironment. Nucleic Acids Res..

[30] Kang Y.-J., Pan L., Liu Y., Rong Z., Liu J., Liu F. (2025). GEPIA3: Enhanced Drug Sensitivity and Interaction Network Analysis for Cancer Research. Nucleic Acids Res..

[31] Lonsdale J., Thomas J., Salvatore M., Phillips R., Lo E., Shad S., Hasz R., Walters G., Garcia F., Young N. (2013). The Genotype-Tissue Expression (GTEx) Project. Nat. Genet..

[32] Chandrashekar D.S., Karthikeyan S.K., Korla P.K., Patel H., Shovon A.R., Athar M., Netto G.J., Qin Z.S., Kumar S., Manne U. (2022). UALCAN: An Update to the Integrated Cancer Data Analysis Platform. Neoplasia.

[33] Cui H., Zhao G., Lu Y., Zuo S., Duan D., Luo X., Zhao H., Li J., Zeng Z., Chen Q. (2025). TIMER3: An Enhanced Resource for Tumor Immune Analysis. Nucleic Acids Res..

[34] Heath A.P., Ferretti V., Agrawal S., An M., Angelakos J.C., Arya R., Bajari R., Baqar B., Barnowski J.H.B., Burt J. (2021). The NCI Genomic Data Commons. Nat. Genet..

[35] Gao J., Aksoy B.A., Dogrusoz U., Dresdner G., Gross B., Sumer S.O., Sun Y., Jacobsen A., Sinha R., Larsson E. (2013). Integrative Analysis of Complex Cancer Genomics and Clinical Profiles Using the cBioPortal. Sci. Signal..

[36] Cerami E., Gao J., Dogrusoz U., Gross B.E., Sumer S.O., Aksoy B.A., Jacobsen A., Byrne C.J., Heuer M.L., Larsson E. (2012). The cBio Cancer Genomics Portal: An Open Platform for Exploring Multidimensional Cancer Genomics Data. Cancer Discov..

[37] Liu J., Lichtenberg T., Hoadley K.A., Poisson L.M., Lazar A.J., Cherniack A.D., Kovatich A.J., Benz C.C., Levine D.A., Lee A.V. (2018). An Integrated TCGA Pan-Cancer Clinical Data Resource to Drive High-Quality Survival Outcome Analytics. Cell.

[38] Ceccarelli M., Barthel F.P., Malta T.M., Sabedot T.S., Salama S.R., Murray B.A., Morozova O., Newton Y., Radenbaugh A., Pagnotta S.M. (2016). Molecular Profiling Reveals Biologically Discrete Subsets and Pathways of Progression in Diffuse Glioma. Cell.

[39] Liu X., Li Y., Qian Z., Sun Z., Xu K., Wang K., Liu S., Fan X., Li S., Zhang Z. (2018). A Radiomic Signature as a Non-Invasive Predictor of Progression-Free Survival in Patients with Lower-Grade Gliomas. NeuroImage Clin..

[40] Zhang K., Liu X., Li G., Chang X., Li S., Chen J., Zhao Z., Wang J., Jiang T., Chai R. (2022). Clinical Management and Survival Outcomes of Patients with Different Molecular Subtypes of Diffuse Gliomas in China (2011–2017): A Multicenter Retrospective Study from CGGA. Cancer Biol. Med..

[41] Wang Y., Qian T., You G., Peng X., Chen C., You Y., Yao K., Wu C., Ma J., Sha Z. (2015). Localizing Seizure-Susceptible Brain Regions Associated with Low-Grade Gliomas Using Voxel-Based Lesion-Symptom Mapping. Neuro-Oncol..

[42] Gu Z. (2022). Complex Heatmap Visualization. iMeta.

[43] Wickham H. (2016). Ggplot2: Elegant Graphics for Data Analysis.

[44] Therneau T.M. (2026). A Package for Survival Analysis in R. Version 3.8-6. https://cran.r-project.org/web/packages/survival.

[45] Kassambara A., Kosinski M., Biecek P. (2026). Survminer: Drawing Survival Curves Using “ggplot2”, Version 0.5.2. CRAN Contrib. Packages. https://cran.r-project.org/web/packages/survminer/index.html.

[46] Blanche P., Dartigues J.-F., Jacqmin-Gadda H. (2013). Estimating and Comparing Time-Dependent Areas under Receiver Operating Characteristic Curves for Censored Event Times with Competing Risks. Stat. Med..

[47] Ritchie M.E., Phipson B., Wu D., Hu Y., Law C.W., Shi W., Smyth G.K. (2015). Limma Powers Differential Expression Analyses for RNA-Sequencing and Microarray Studies. Nucleic Acids Res..

[48] Wu T., Hu E., Xu S., Chen M., Guo P., Dai Z., Feng T., Zhou L., Tang W., Zhan L. (2021). clusterProfiler 4.0: A Universal Enrichment Tool for Interpreting Omics Data. Innovation.

[49] Carlson M. (2025). Org.Hs.Eg.Db. Genome Wide Annotation for Human, Version 3.22.0; Bioconductor. https://bioconductor.org/packages/release/data/annotation/html/org.Hs.eg.db.html.

[50] Dolgalev I. (2026). R Package.

[51] Liberzon A., Birger C., Thorvaldsdóttir H., Ghandi M., Mesirov J.P., Tamayo P. (2015). The Molecular Signatures Database (MSigDB) Hallmark Gene Set Collection. Cell Syst..

[52] Yu G. (2026). R Package.

[53] Newman A.M., Liu C.L., Green M.R., Gentles A.J., Feng W., Xu Y., Hoang C.D., Diehn M., Alizadeh A.A. (2015). Ro-bust Enumeration of Cell Subsets from Tissue Expression Profiles. Nat. Methods.

[54] Hänzelmann S., Castelo R., Guinney J. (2013). GSVA: Gene Set Variation Analysis for Microarray and RNA-Seq Data. BMC Bioinform..

[55] Margan M., Falcon S., Gentleman R. (2025). R Package.

[56] Becht E., Giraldo N.A., Lacroix L., Buttard B., Elarouci N., Petitprez F., Selves J., Laurent-Puig P., Sautès-Fridman C., Fridman W.H. (2016). Estimating the Population Abundance of Tissue-Infiltrating Immune and Stromal Cell Populations Using Gene Expression. Genome Biol..

[57] Joseph J.V., Conroy S., Tomar T., Eggens-Meijer E., Bhat K., Copray S., Walenkamp A.M.E., Boddeke E., Balasubramanyian V., Wagemakers M. (2014). TGF-β Is an Inducer of ZEB1-Dependent Mesenchymal Transdifferentiation in Glioblastoma That Is Associated with Tumor Invasion. Cell Death Dis..

[58] Chanoch-Myers R., Wider A., Suva M.L., Tirosh I. (2022). Elucidating the Diversity of Malignant Mesenchymal States in Glioblastoma by Integrative Analysis. Genome Med..

[59] Khasraw M., Reardon D.A., Weller M., Sampson J.H. (2020). PD-1 Inhibitors: Do They Have a Future in the Treatment of Glioblastoma?. Clin. Cancer Res..

[60] Liu Y., Zhou F., Ali H., Lathia J.D., Chen P. (2024). Immunotherapy for Glioblastoma: Current State, Challenges, and Future Perspectives. Cell. Mol. Immunol..

[61] Roy L.-O., Poirier M.-B., Fortin D. (2015). Transforming Growth Factor-Beta and Its Implication in the Malignancy of Gliomas. Target. Oncol..

[62] Qazi S., Potts M., Myers S., Richardson S., Trieu V. (2025). Positive Prognostic Overall Survival Impacts of Methylated TGFB2 and MGMT in Adult Glioblastoma Patients. Cancers.

[63] Trieu V., Maida A.E., Qazi S. (2024). Transforming Growth Factor Beta 2 (TGFB2) mRNA Levels, in Conjunction with Interferon-Gamma Receptor Activation of Interferon Regulatory Factor 5 (IRF5) and Expression of CD276/B7-H3, Are Therapeutically Targetable Negative Prognostic Markers in Low-Grade Gliomas. Cancers.

[64] Youssef G., Aquilanti E., Miller J.J., Lan Z., Lasica A.B., Arrillaga-Romany I., Batchelor T.T., Berger T.R., Beroukhim R., Chukwueke U. (2026). Prognostic Significance of O6-Methylguanine-DNA Methyltransferase Promoter Methylation Status in Isocitrate Dehydrogenase-Mutant Glioma. Neuro-Oncol..

[65] Sharma P., Hu-Lieskovan S., Wargo J.A., Ribas A. (2017). Primary, Adaptive, and Acquired Resistance to Cancer Immunotherapy. Cell.

[66] Chen D.S., Mellman I. (2017). Elements of Cancer Immunity and the Cancer–Immune Set Point. Nature.

[67] Neftel C., Laffy J., Filbin M.G., Hara T., Shore M.E., Rahme G.J., Richman A.R., Silverbush D., Shaw M.L., Hebert C.M. (2019). An Integrative Model of Cellular States, Plasticity, and Genetics for Glioblastoma. Cell.

[68] Hara T., Chanoch-Myers R., Mathewson N.D., Myskiw C., Atta L., Bussema L., Eichhorn S.W., Greenwald A.C., Kinker G.S., Rodman C. (2021). Interactions between Cancer Cells and Immune Cells Drive Transitions to Mesenchymal-like States in Glioblastoma. Cancer Cell.

[69] Wang Q., Hu B., Hu X., Kim H., Squatrito M., Scarpace L., deCarvalho A.C., Lyu S., Li P., Li Y. (2017). Tumor Evolution of Glioma-Intrinsic Gene Expression Subtypes Associates with Immunological Changes in the Microenvironment. Cancer Cell.

[70] Zhou X., Jin G., Zhang J., Liu F. (2023). Recruitment Mechanisms and Therapeutic Implications of Tumor-Associated Macrophages in the Glioma Microenvironment. Front. Immunol..

[71] Butovsky O., Weiner H.L. (2018). Microglial Signatures and Their Role in Health and Disease. Nat. Rev. Neurosci..

[72] Hambardzumyan D., Gutmann D.H., Kettenmann H. (2016). The Role of Microglia and Macrophages in Glioma Maintenance and Progression. Nat. Neurosci..

[73] Jackson C., Cherry C., Bom S., Dykema A.G., Wang R., Thompson E., Zhang M., Li R., Ji Z., Hou W. (2025). Distinct Myeloid-Derived Suppressor Cell Populations in Human Glioblastoma. Science.

[74] Greenwald A.C., Darnell N.G., Hoefflin R., Simkin D., Mount C.W., Gonzalez Castro L.N., Harnik Y., Dumont S., Hirsch D., Nomura M. (2024). Integrative Spatial Analysis Reveals a Multi-Layered Organization of Glioblastoma. Cell.

[75] Haddad A.F., Young J.S., Oh J.Y., Okada H., Aghi M.K. (2022). The Immunology of Low-Grade Gliomas. Neurosurg. Focus.

[76] Yearley J.H., Gibson C., Yu N., Moon C., Murphy E., Juco J., Lunceford J., Cheng J., Chow L.Q.M., Seiwert T.Y. (2017). PD-L2 Expression in Human Tumors: Relevance to Anti-PD-1 Therapy in Cancer. Clin. Cancer Res..

[77] Zhou Y., Yao L., Ma T., Wang Z., Yin Y., Yang J., Zhang X., Zhang M., Qin G., Ma J. (2024). Indoleamine 2,3-Dioxygenase-1 Involves in CD8+T Cell Exhaustion in Glioblastoma via Regulating Tryptophan Levels. Int. Immunopharmacol..

[78] Khasraw M., Weller M., Lorente D., Kolibaba K., Lee C.K., Gedye C., I de La Fuente M., Vicente D., Reardon D.A., Gan H.K. (2021). Bintrafusp Alfa (M7824), a Bifunctional Fusion Protein Targeting TGF-β and PD-L1: Results from a Phase I Expansion Cohort in Patients with Recurrent Glioblastoma. Neuro-Oncol. Adv..

[79] Li N., Rodriguez J.L., Yin Y., Logun M.T., Zhang L., Yu S., Hicks K.A., Zhang J.V., Zhang L., Xie C. (2024). Armored Bicistronic CAR T Cells with Dominant-Negative TGF-β Receptor II to Overcome Resistance in Glioblastoma. Mol. Ther..

[80] Metropulos A.E., Munshi H.G., Principe D.R. (2022). The Difficulty in Translating the Preclinical Success of Combined TGFβ and Immune Checkpoint Inhibition to Clinical Trial. eBioMedicine.

