# Supplementary figures and images for: TGFB2 as a Prognostic Biomarker Associated with Myeloid-Enriched, Multi-Checkpoint-Activated Immunosuppression in Diffuse Glioma: A Multi-Cohort Transcriptomic Study
Source: Cancers (Basel). 2026 Jun 27;18(13):2092. doi: 10.3390/cancers18132092 (PMC13359701; doi:10.3390/cancers18132092)

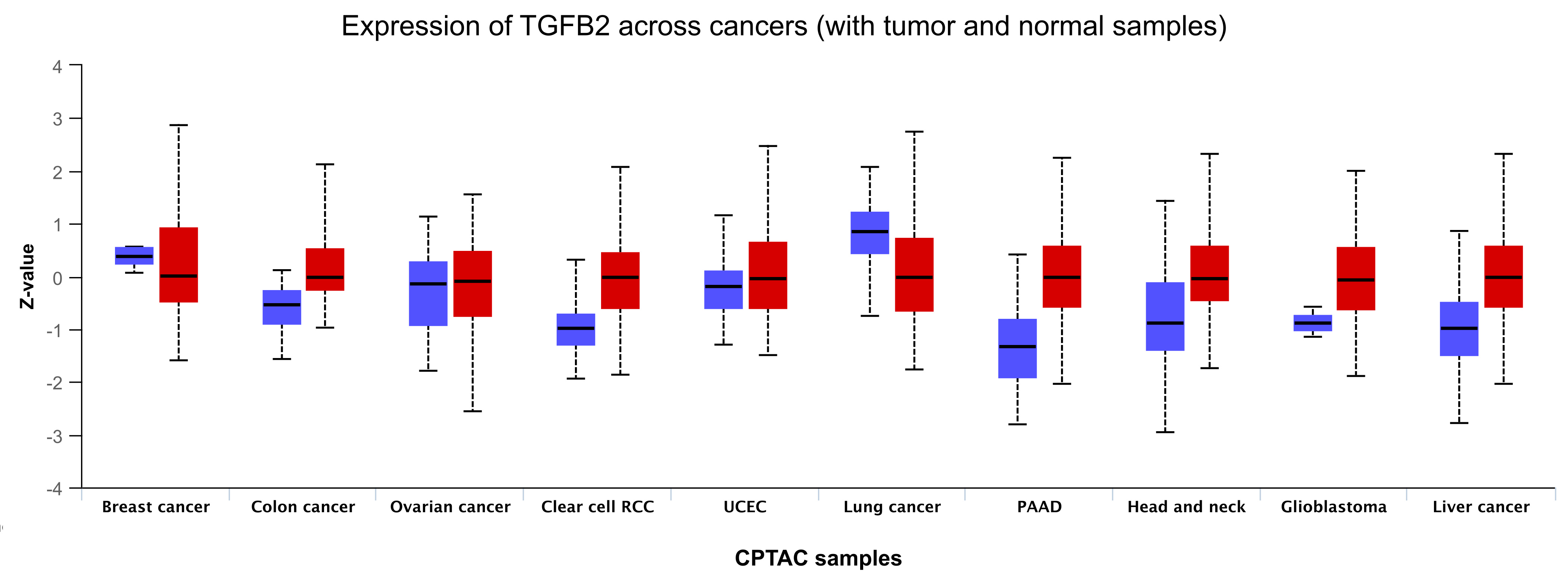

Supplement: Supplementary file 1 [file cancers-18-02092-s001.zip › Supplementary Figure S1_Pan-cancer CPTAC proteomic profiling of TGFB2.tiff]

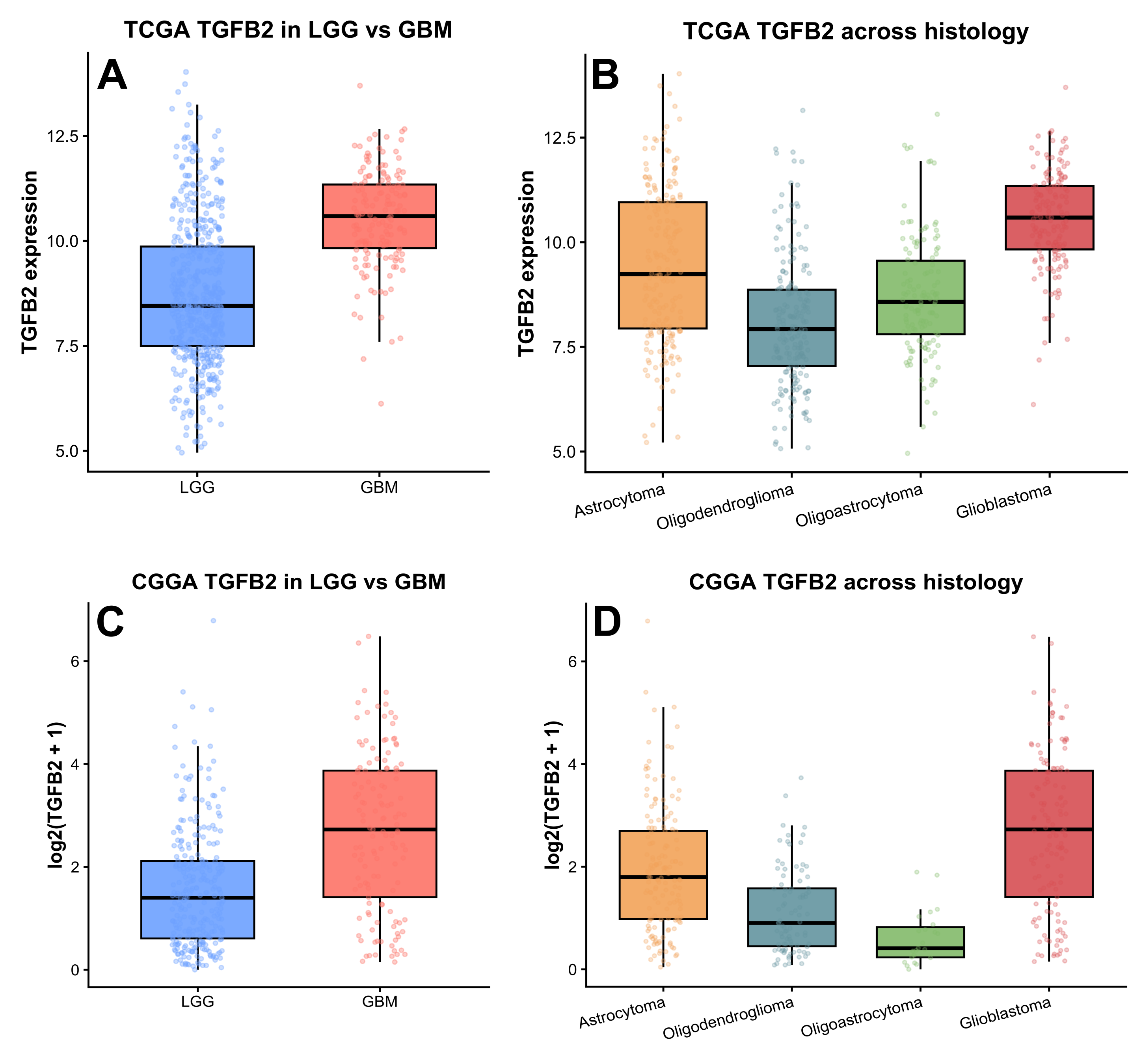

Supplement: Supplementary file 1 [file cancers-18-02092-s001.zip › Supplementary Figure S2_TGFB2 expression across clinicopathological subgroups in TCGA and CGGA.tiff]

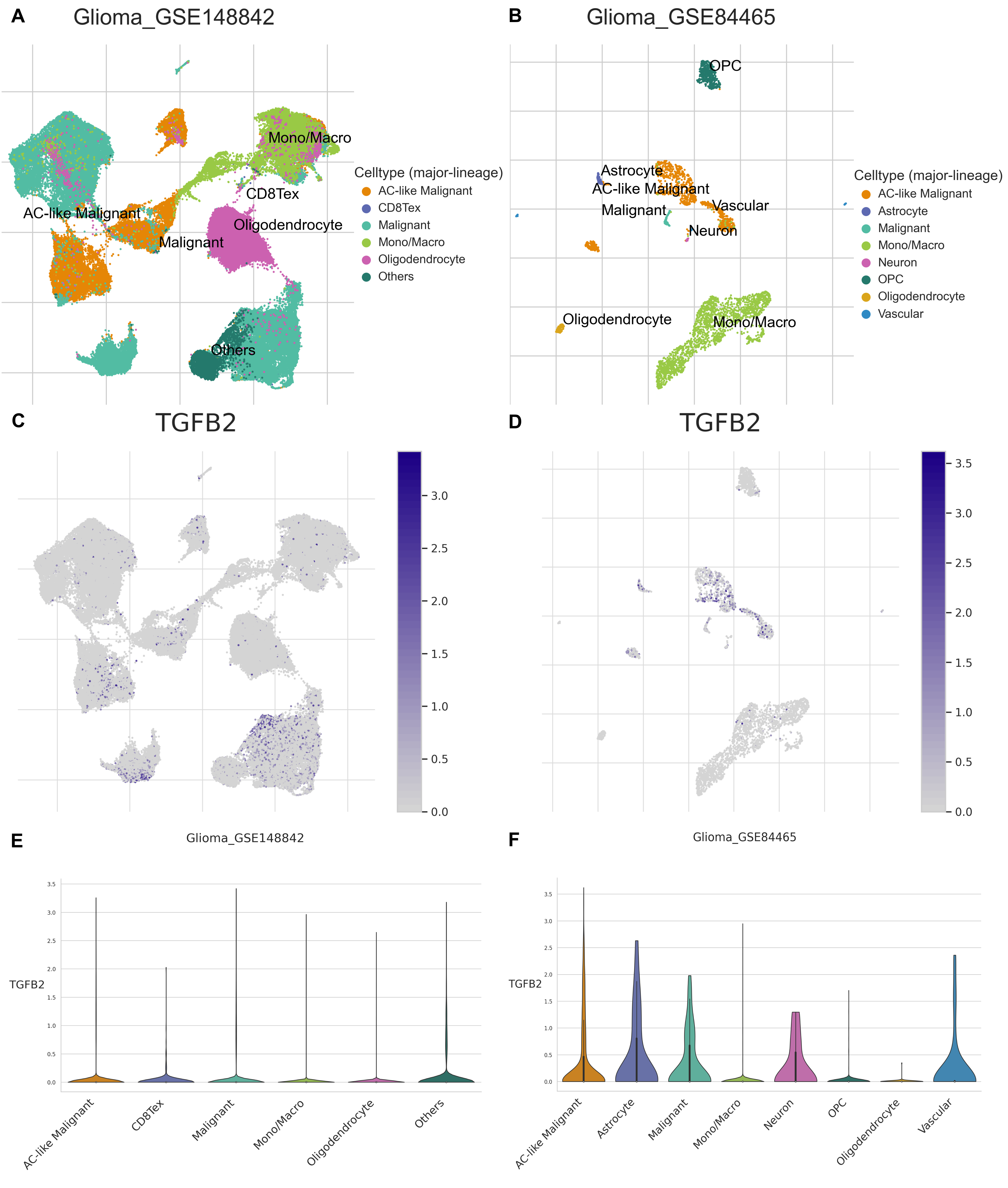

Supplement: Supplementary file 1 [file cancers-18-02092-s001.zip › Supplementary Figure S6_Single-cell TGFB2 expression in representative GBM datasets from TISCH2.tiff]
